# Eco-Friendly Syntheses of 2-Substituted Benzoxazoles and 2-Substituted Benzothiazoles from 2-Aminophenols, 2-Aminothiophenols and DMF Derivatives in the Presence of Imidazolium Chloride

**DOI:** 10.3390/molecules24010174

**Published:** 2019-01-04

**Authors:** Qingqiang Tian, Wen Luo, Zongjie Gan, Dan Li, Zeshu Dai, Huajun Wang, Xuetong Wang, Jianyong Yuan

**Affiliations:** Department of Medicinal Chemistry, College of Pharmacy, Chongqing Medical University, Chongqing 400016, China; Tqingqiang@163.com (Q.T.); Lwen1129@163.com (W.L.); gzj@cqmu.edu.cn (Z.G.); ld28115@163.com (D.L.); daizs11@163.com (Z.D.); Wanghuajun333@163.com (H.W.); wxy998298@163.com (X.W.)

**Keywords:** imidazolium chloride, benzoxazoles and benzothiazoles derivatives, syntheses

## Abstract

A simple, economical and metal-free approach to the synthesis of 2-substituted benzoxazoles and 2-substituted benzothiazoles from 2-aminophenols, 2-aminothiophenols and DMF derivatives, only using imidazolium chloride (50% mmol) as promoter without any other additive, was reported. Various 2-substituted benzoxazoles and 2-substituted benzothiazoles were thus prepared in moderate to excellent yields.

## 1. Introduction

**Figure 1:**
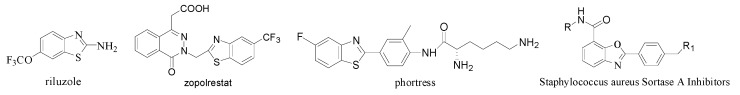
Biologically active benzoxazole and benzothiazole derivatives.

The 2-substituted benzoxazoles and 2-substituted benzothiazoles are well-known substructures in a variety of biologically active natural compounds [1,2], photoluminescent materials [3,4] or pharmaceutical agents such as riluzole, a treatment for amyotrophic lateral sclerosis [5], zopolrestat, an aldose reductase inhibitor used for the treatment of diabetes mellitus [6], the antitumor agent phortress, which acts via binding to aryl hydrocarbon receptors [7], 2-phenylbenzo[d]oxazole-7-carboxamide derivatives which are potential *Staphylococcus aureus sortase A Inhibitors* [8], and so on [9,10] (Figure 1).

Because of this importance, much effort has been devoted to the development of synthetic methods for the synthesis of benzoxazole and benzothiazole derivatives. Several methods developed to synthesize 2-substituted and unsubstituted benzoxazoles or benzothiazoles via the condensation *O*-aminophenols/-thiophenols with aldehydes [11], acids [12], amides [13], acyl chlorides [14], nitriles [15] or esters [16], as well as cyclization of thiobenzanilides have been reported [17,18,19,20]. 

**Scheme 1:**
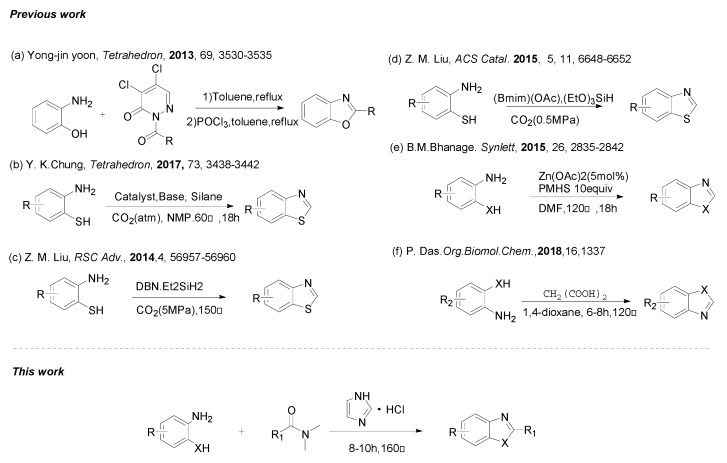
Syntheses of benzoxazole and benzothiazole derivatives.

Since these reports, many improvements to these reactions have been made by the use of alternative catalysts. In 2013, Yoon et al. [21] reported an improved method to form benzimidazoles using 2-acyl-4,5-dichloropyridazin-3(2*H*)-one as acyl transfer agent under transition-metal-free conditions, (Scheme 1a), but, this reagent is expensive and additional POCl_3_ is needed. Liu and Chung have reported [22,23,24] the synthesis of benzothiazoles from 2-aminobenzenethiol using silane as a CO_2_ fixing agent. However, the practicality of these methods is offset by the need for a special reactor, the stoichiometric amount of catalyst and limited substrate scope which diminish their synthetic utility in practical application (Scheme 1b–d). Bhanage et al. [25] have reported an improved method to form benzimidazoles from *O*-phenylenediamines and DMF. In their reports, a metal catalyst (Zn(OAc)_2_) was employed (Scheme 1e). Recently, Das et al. [26] reported oxalic/malonic acids as carbon building blocks for benzoxazole, quinazoline and quinazolinone synthesis. In their reports, a large amount (4–8 equiv.) of corrosive oxalic/malonic acids were employed (Scheme 1f).

**Scheme 2:**
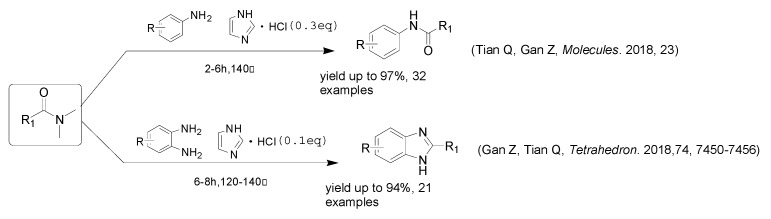
Previously explored and reported routes.

However, most of these methods suffer from one or more disvantages, including the use of some environmental unfriendly catalysts, the use of expensive reagents, long reaction times, toxic or non-reusable catalysts, metal catalysts and additional reagents. Therefore, efficient and environmentally friendly syntheses of 2-substituted benzoxazoles and 2-substituted benzothiazoles heterocycles are of great importance. We have recently reported [27,28] the use of imidazolium chloride as catalyst and DMF derivatives as eco-friendly carbon sources in the formation of amido bonds and benzimidazole derivatives (Scheme 2). We envisioned that this process might be extended to 2-aminophenols and 2-aminothiophenols, which would provide access to benzoxazole and benzothiazole derivatives using inexpensive, stable, and easy to synthesize DMF derivatives as carbon sources and imidazolium chloride as the promoter [29,30,31]. 

## 2. Results and Discussion

Considering the imidazolium chloride catalyst system has been successfully applied in the formation of amido bonds and benzimidazole derivatives, next, we attempted to synthesize 2-substituted benzoxazole derivatives by reacting *O*-aminophenol with DMF derivatives under the same conditions, however, the results showed that the main products were acetamide intermediates due to the less reactivity of the hydroxyl group compared to the amino group under these conditions (Table 1, entries 1–4).

The results of our experiments to optimize the reaction conditions for the synthesis of 2-substituted benzoxazoles, using 2-aminophenol (**1a**) as a model substrate, are listed in Table 2.

Initially, the reaction did not occur in DMA without imdazolium chloride (Table 2, entry 1) and 0.5 equiv. of HCl was found to give only traces of product **2a** (Table 2, entry 2). The reaction was performed in DMA at 140 °C and we obtained a 18% yield of the product (Table 2, entry 3). Furthermore, on increasing the temperature to 150 and 160 °C for 8 h the product was obtained in 43% and 60% yield, respectively (Table 2, entries 4, 5), indicating that the reaction was highly sensitive to temperature. Encouraged by this promising result, next, with the aim to decrease the imidazolium chloride loading, we then carried out the reaction at 160 °C. It was observed that under these conditions 0.5 equiv. of imidazolium chloride were sufficient to obtain a good yield, and no further improvements in the yield of the target product were observed with higher mole equivalents of imidazolium chloride (Table 2, entries 8, 9 and 10). In addition, we tried various solvents like xylenes, water and benzene, but unsatisfactory yields of the product was obtained (Table 2, entries 11, 12 and 13).

Under the optimized conditions, we next set out to examine the scope and limitations of this reaction and the results are shown in Table 3. All the substrates listed afforded the corresponding target product, under the standard conditions, in moderate to good yield (52–87%). Both electron-donating and electron-withdrawing groups were tolerated under the reaction conditions. Mono-substituents on the benzene moiety showed no obvious influence on the reaction outcome and produced desired products in good yields (Table 3, **2a, 2c, 2g** and **2h**). It is noteworthy to mention that a functional group like bromo (Table 3, **2h**) is tolerable under this condition. With aromatic-substituted substrates, the reaction affords the corresponding benzoxazole derivatives **2b**–**f**, **2d** and **2g** in 52–86% yields, respectively (Table 2, entries 2, 3, 4, 7 and 9), although a longer reaction time is required. However, when the phenyl ring bore electron-withdrawing substituents, such as a nitro group (Table 3, **2f**), the yield was decreased. These results suggest that the electron-withdrawing groups disfavor the formation of the activated reaction intermediate.

Next, to evaluate further the scope of this methodology, the reaction was carried out with a series of *O*-aminothiophenol and DMF derivatives, affording the corresponding target products in moderate to good yields (Table 4), under the same optimized conditions. 

**Scheme 3:**
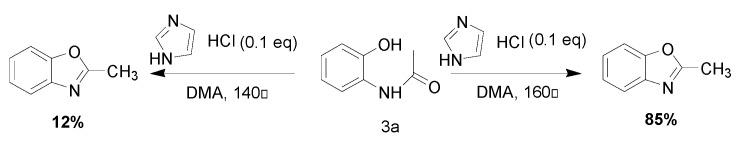
Control experiments to elucidate the mechanism.

Different *para* substituents on the *O*-aminothiophenols **3a**–**d** also gave good yields (75–85%, Table 4, **4a–4k**). Similarly, the cyclization reactions of *O*-aminophenol with aromatic-substituted DMF derivatives also gave the target products **4e**–**4k** in excellent yields. 2-amino-5-nitrobenzenethiol and *N*,*N*-Dimethyl-4-nitrobenzamide acid dimethylamide substrate were also found to be stabilized under the conditions and gave the desired products **4d** and **4g** in moderate yields (75%, 60% and 75%). In addition, a heteroaromatic substrate was well tolerated and gave the desired target product **4j** in 75% yield. To explore the reaction mechanism, control experiments were conducted under the standard reaction conditions. By stirring *N*-(2-hydroxyphenyl)-acetamide with DMA at 140 °C for 8 h in the presence of 0.1 eq imidazolium chloride only 12% yield of target product was obtained (Scheme 3), while a satisfactory yield (85%) was observed at 160 °C, indicating that the reaction was highly sensitive to temperature. 

**Scheme 4:**
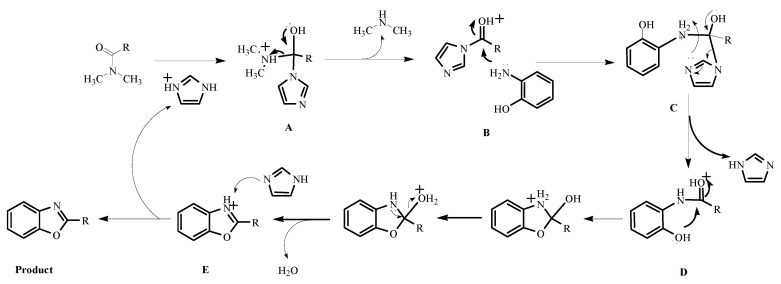
A plausible mechanism for the imidazolium chloride-promoted cyclization.

Therefore, based on these experiment results and literature [27,28,32], a plausible mechanism for the formation of benzoxazoles is proposed in Scheme 4. First, DMA is activated by imidazolium chloride to afford the tetrahedral intermediate A. Breakdown of tetrahedral intermediate A leads to the formation of intermediate B (*N*-acylimidazole) and dimethylamine gas. Next the intermediate B reacts with *O*-aminophenol to form compound C, followed by extrusion of one molecule of imidazole to generate compound D. Afterwards, the intermediate E was formed by intramolecular cyclization of compound D, and one molecule of H_2_O was eliminated. Finally, intermediate E furnishes the target product, recycling the imidazole chloride.

## 3. Conclusions

In conclusion, in the present work, we have reported a new, efficient, metal free and economical method to synthesize 2-substituted benzoxazoles and 2-substituted benzothiazoles from 2-amino-phenols or 2-aminothiophenols and DMF derivatives in the presence of imidazolium chloride without any other catalysts or additives. This method has wide substrate scope, providing moderate to excellent yields of the target products. Further applications of imidazolium chloride in the synthesis of other heterocycles or important medical intermediates are currently under investigation in our laboratory.

## 4. Experimental

### 4.1. General Information

All reagents were purchased from Ltd. (Shenzhen, China), Meyer Reagent Co., Ltd. (Shanghai, China), Macklin Reagent Co., Ltd. (Shanghai, China), Chongqing Chuandong Chemical Co., Ltd. (Chongqing, China). etc., and used without further purification. ^1^H- (600 MHz) and ^13^C-NMR (151 MHz) spectra were recorded on an Avance III NMR spectrometer (Bruker, Bruker, Fällande, Switzerland) in CDCl_3_ using tetramethylsilane (TMS) or the residual CHCl_3_ signals as internally reference. Chemical shifts are reported in ppm and coupling constants (*J*) in Hz. Open-bed chromatography was carried out on silica gel (200–300 mesh, Qingdao, China) using gravity flow. All substrates are known compounds according to the literature.

### 4.2. General Procedure for the Synthesis of Benzoxazole Derivatives ***2a**–**2d***

A mixture of **1a** (0.6 g, 5.5 mmol, 1 equiv.), imidazolium chloride (0.17 g, 1.65 mmol, 0.3 equiv.) and DMA (5 mL) was stirred at 140 °C for 8 h. When the reaction was completed, water (15 mL) and ethyl acetate (20 mL) were added with stirring to the reaction mixture. The organic layer was extracted and dried over anhydrous Na_2_SO_4_, filtered and concentrated under reduced pressure. The resulting residue was purified by column chromatography on silica gel using PE/EA as eluent to give the target product **2a**. 

### 4.3. General Procedure for the Synthesis of Benzoxazole Derivatives ***2a***, ***2c***, ***2g*** and ***2h*** and Benzothiazole Derivatives ***4a**–**4d***

A tube-type Schlenk flask was charged with **1a** (0.6 g, 5.5 mmol, 1 equiv.), imidazolium chloride (0.28 g, 2.75 mmol, 0.5 equiv.) and DMA (5 mL) and the mixture was stirred at 160 °C for 8 h. When the reaction was complete water (15 mL) and ethyl acetate (20 mL) were added with stirring to the reaction mixture. The organic layer was extracted and dried over anhydrous Na_2_SO_4_, filtered and concentrated under reduced pressure. The resulting residue was purified by column chromatography on silica gel using PE/EA as eluent to give the corresponding product **4a**.

### 4.4. General Procedure for the Synthesis of Benzoxazole Derivatives ***2b***, ***2d***, ***2e***, ***2f***, ***2i*** and Benzothiazole Derivatives ***4e**–**4k***

To a mixture of **1a** (0.6 g, 5.5 mmol, 1 equiv.), imidazolium chloride (0.25 g, 2.3 mmol, 0.5 equiv.) and *N*,*N*-dimethylbenzamide (1.43 g, 11.0 mmol, 2 equiv.) was added. The mixture was stirred at 160 °C for 10 h. After completion of the reaction water (15 mL) was added and the resulting mixture was extracted thrice with EA (20 mL), and the combined organic layers were dried over anhydrous Na_2_SO_4_ and concentrated. The residue was purified by column chromatography on silica gel using PE/EA as eluent to obtain the pure desired product.

### 4.5. Product Characterization Data 

*2-Methylbenzo[d]oxazole* (**2a**) [33]: The product was obtained as yellow liquid in 80% yield. ^1^H-NMR δ 7.64 (d, *J* = 7.2 Hz, 1H), 7.45 (d, *J* = 7.6 Hz, 1H), 7.29–7.25 (m, 2H), 2.61 (s, 3H). ^13^C-NMR δ 163.74, 150.94, 141.49, 124.39, 124.04, 119.38, 110.15, 14.46. (See Supplementary Materials).

*2-Phenylbenzo[d]oxazole* (**2b**) [34]: The product was obtained as white solid in 86% yield. m.p.: 101–103 °C. ^1^H-NMR δ 8.27 (d, *J* = 6.3 Hz, 2H), 7.80–7.78 (m, 1H), 7.60–7.58 (m, 1H), 7.53 (d, *J* = 7.0 Hz, 3H), 7.36 (dd, *J* = 6.0, 3.1 Hz, 2H). ^13^C-NMR δ 162.02, 149.72, 140.96, 130.55, 127.90, 126.63, 126.07, 124.12, 123.59, 118.97, 109.58. (See Supplementary Materials).

*2,6-Dimethylbenzo[d]oxazole* (**2c**) [35]: The product was obtained as yellow liquid in 86% yield. ^1^H-NMR δ 7.42 (d, *J* = 8.1 Hz, 1H), 7.17 (s, 1H), 7.01 (d, *J* = 8.0 Hz, 1H), 2.51 (s, 3H), 2.37 (s, 3H). ^13^C-NMR δ 163.23, 151.26, 139.25, 134.71, 125.22, 118.70, 110.39, 21.63, 14.46. (See Supplementary Materials).

*6-Methyl-2-phenylbenzo[d]oxazole* (**2d**) [36]: The product was obtained as yellow solid in 83% yield. m.p.: 90–92 °C. ^1^H-NMR δ 8.17–8.15 (m, 1H), 7.57 (d, *J* = 8.1 Hz, 1H), 7.44 (d, *J* = 1.6 Hz, 1H), 7.31 (s, 1H), 7.10 (d, *J* = 8.1 Hz, 0H), 2.43 (s, 2H). ^13^C-NMR δ 161.55, 150.03, 138.87, 134.56, 130.26, 127.85, 126.44, 126.32, 124.79, 118.31, 109.74, 20.78. (See Supplementary Materials).

*2-(4-Methoxyphenyl)benzo[d]oxazole* (**2e**) [37]: The product was obtained as white solid in 88% yield. m.p.: 97–100 °C. ^1^H-NMR δ 8.20 (d, *J* = 8.5 Hz, 2H), 7.74 (d, *J* = 8.7 Hz, 1H), 7.56 (d, *J* = 6.8 Hz, 1H), 7.32 (h, *J* = 6.7, 6.1 Hz, 2H), 7.03 (d, *J* = 8.3 Hz, 2H), 3.89 (s, 3H).^13^C-NMR δ 163.17, 162.34, 150.66, 142.21, 129.42, 124.61, 124.43, 119.63, 114.37, 110.39, 55.47. (See Supplementary Materials).

*2-(4-Nitrophenyl)benzo[d]oxazole* (**2f**) [38]: The product was obtained as yellow solid in 52% yield. m.p.: 166–167 °C.^1^H-NMR δ 7.74 (d, *J* = 6.9 Hz, 2H), 7.52 (t, *J* = 7.5 Hz, 2H), 7.42 (t, *J* = 7.8 Hz, 4H), 7.30–7.26 (m, 1H). ^13^C-NMR δ 160.67, 151.04, 149.42, 141.90, 132.80, 128.42, 126.37, 125.25, 124.25, 120.70, 110.96. (See Supplementary Materials).

*2,5-Dimethylbenzo[d]oxazole* (**2g**) [39]: The product was obtained as yellow liquid in 84% yield. ^1^H-NMR δ 7.42 (s, 1H), 7.30 (d, *J* = 22.9 Hz, 1H), 7.07 (d, *J* = 7.6 Hz, 1H), 2.60 (s, 3H), 2.44 (s, 3H). ^13^C-NMR δ 163.85, 149.19, 141.68, 133.82, 125.43, 119.34, 109.51, 21.38, 14.49. (See Supplementary Materials).

*5-Bromo-2-methylbenzo[d]oxazole* (**2h**) [40]: The product was obtained as yellow liquid in 87% yield. ^1^H-NMR δ 7.78 (d, *J* = 1.9 Hz, 1H), 7.39 (d, *J* = 8.6 Hz, 1H), 7.33 (d, *J* = 8.5 Hz, 1H), 2.64 (s, 3H). ^13^C-NMR δ 165.10, 149.94, 143.11, 127.45, 122.46, 116.83, 111.42, 14.56. (See Supplementary Materials).

*5-Bromo-2-phenylbenzo[d]oxazole* (**2i**) [41]: The product was obtained as white solid in 80% yield. m.p.: 108–110 °C. ^1^H-NMR δ 8.24 (d, *J* = 7.5 Hz, 2H), 7.91 (s, 1H), 7.58–7.52 (m, 3H), 7.46 (s, 2H).^13^C-NMR δ 164.16, 149.74, 143.64, 131.97, 128.99, 128.10, 127.78, 126.60, 122.96, 117.33, 111.81. (See Supplementary Materials).

*2-Methylbenzo[d]thiazole* (**4a**) [42]: The product was obtained as yellow liquid in 82% yield. ^1^H-NMR δ 7.95 (d, *J* = 8.0 Hz, 1H), 7.80 (d, *J* = 7.3 Hz, 1H), 7.43 (t, *J* = 7.7 Hz, 1H), 7.34–7.31 (m, 1H), 2.81 (s, 3H). ^13^C-NMR δ 166.90, 153.36, 135.64, 125.90, 124.68, 122.37, 121.38, 20.09. (See Supplementary Materials).

*6-Chloro-2-methylbenzo[d]thiazole* (**4b**) [43]: The product was obtained as yellow solid in 85% yield. m.p.: 79–82 °C ^1^H-NMR δ 7.80 (d, *J* = 8.6 Hz, 1H), 7.74 (s, 1H), 7.35 (d, *J* = 8.6 Hz, 1H), 2.78 (s, 3H). ^13^C-NMR δ 167.78, 151.49, 136.64, 130.79, 126.85, 123.05, 121.08, 20.13. (See Supplementary Materials).

*5-Chloro-2-methylbenzo[d]thiazole* (**4c**) [43]: The product was obtained as white solid in 80% yield. m.p.: 60–62 °C. ^1^H-NMR δ 7.93 (d, *J* = 2.0 Hz, 1H), 7.73 (d, *J* = 8.5 Hz, 1H), 7.33 (d, *J* = 6.5 Hz, 1H), 2.84 (s, 3H). ^13^C-NMR δ 169.04, 154.13, 133.85, 132.00, 125.24, 122.29, 122.09, 20.23. (See Supplementary Materials).

*2-Methyl-6-nitrobenzo[d]thiazole* (**4d**) [44]: The product was obtained as yellow solid in 75% yield. m.p.: 161–162 °C. ^1^H-NMR δ 8.78 (s, 1H), 8.34 (d, *J* = 6.7 Hz, 1H), 8.04 (d, *J* = 8.9 Hz,1H), 2.93 (s, 3H). ^13^C-NMR δ 173.30, 157.09, 144.81, 136.02, 122.65, 121.59, 118.01, 20.71. (See Supplementary Materials).

*2-(4-Methoxyphenyl)benzo[d]thiazole* (**4e**) [21]: The product was obtained as white solid in 87% yield. m.p.: 119–120 °C. ^1^H-NMR δ 8.04 (d, *J* = 8.6 Hz, 3H), 7.87 (d, *J* = 7.6 Hz, 1H), 7.47 (t, *J* = 7.4 Hz, 1H), 7.35 (t, *J* = 7.6 Hz, 1H), 7.00 (d, *J* = 8.8 Hz, 2H), 3.87 (s, 3H).^13^C-NMR δ 167.91, 161.96, 154.11, 134.80, 129.15, 126.36, 126.25, 124.83, 122.80, 121.53, 114.57, 114.39, 77.26, 77.13, 55.49. (See Supplementary Materials).

*2-Phenylbenzo[d]thiazole* (**4f**) [21]: The product was obtained as white solid in 79% yield. m.p.: 111–113 °C. ^1^H-NMR δ 8.12–8.08 (m, 3H), 7.91 (d, *J* = 8.0 Hz, 1H), 7.50 (p, *J* = 4.1 Hz, 4H), 7.39 (t, *J* = 7.6 Hz, 1H). ^13^C-NMR δ 168.15, 153.99, 135.00, 133.52, 131.06, 129.07, 127.62, 126.39, 125.26, 123.23, 121.66. (See Supplementary Materials).

*2-(4-Nitrophenyl)benzo[d]thiazole* (**4g**) [45]: The product was obtained as yellow solid in 60% yield. m.p.: 224–226 °C. ^1^H-NMR δ 8.29 (d, *J* = 8.9 Hz, 2H), 8.21 (d, *J* = 8.8 Hz, 2H), 8.07 (d, *J* = 8.2 Hz, 1H), 7.89 (d, *J* = 7.8 Hz, 1H), 7.51–7.48 (m, 1H), 7.42–7.39 (m,1H). ^13^C-NMR δ 163.82, 153.08, 148.02, 138.16, 134.46, 127.23, 125.90, 125.21, 123.30, 122.92, 120.82. (See Supplementary Materials).

*2-(4-Chlorophenyl)benzo[d]thiazole* (**4h**) [46]: The product was obtained as white solid in 82% yield. m.p.: 114–115 °C. ^1^H-NMR) δ 8.07 (d, *J* = 8.2 Hz, 1H), 8.04–8.01 (m, 2H), 7.90 (dd, *J* = 8.0, 1.1 Hz, 1H), 7.50 (ddd, *J* = 8.2, 7.1, 1.2 Hz, 1H), 7.47–7.45 (m, 2H), 7.41–7.38 (m, 1H). ^13^C-NMR δ 166.65, 154.02, 137.06, 135.04, 132.08, 129.29, 128.73, 126.52, 125.45, 123.30, 121.67. (See Supplementary Materials).

*2-(2-Chlorophenyl)benzo[d]thiazole* (**4i**) [46]: The product was obtained as white solid in 79% yield. m.p.: 81–83 °C. ^1^H-NMR δ 8.24–8.19 (m, 1H), 8.16–8.12 (m, 1H), 7.95 (dd, *J* = 8.1, 1.1 Hz, 1H), 7.57–7.51 (m, 2H), 7.47–7.39 (m, 3H). ^13^C-NMR δ 164.20, 152.38, 136.05, 132.71, 132.19, 131.76, 131.17, 130.81, 127.12, 126.32, 125.47, 123.43, 121.40. (See Supplementary Materials).

*2-(Pyridin-2-yl)benzo[d]thiazole* (**4j**) [47]: The product was obtained as white solid in 75% yield. m.p.: 133–135 °C. ^1^H-NMR) δ 8.70 (d, *J* = 4.0 Hz, 1H), 8.39 (d, *J* = 8.0 Hz, 1H), 8.10 (d, *J* = 8.2 Hz, 1H), 7.97 (d, *J* = 7.6 Hz, 1H), 7.86 (t, *J* = 7.7 Hz, 1H), 7.51 (t, *J* = 7.0 Hz, 1H), 7.44–7.38 (m, 2H). ^13^C-NMR δ 169.35, 154.22, 151.37, 149.65, 137.05, 136.11, 126.30, 125.67, 125.29, 123.57, 122.02, 120.81. (See Supplementary Materials).

*3-Benzothiazol-2-yl-1-phenyl-propan-1-one* (**4k**) [48]: The product was obtained as white solid in 82% yield. m.p.: 93–95 °C. ^1^H-NMR δ 8.02 (d, *J* = 7.8 Hz, 2H), 7.95 (d, *J* = 8.1 Hz, 1H), 7.83 (d, *J* = 8.0 Hz, 1H), 7.57 (t, *J* = 7.4 Hz, 1H), 7.50–7.40 (m, 3H), 7.34 (t, *J* = 7.6 Hz, 1H), 3.66 (t, *J* = 7.1 Hz, 2H), 3.57 (t, *J* = 7.1 Hz, 2H). ^13^C-NMR δ 197.87, 170.59, 153.09, 136.50, 135.24, 133.36, 128.69, 128.14, 125.97, 124.85, 122.52, 121.54, 37.64, 28.29. (See Supplementary Materials).

## Figures and Tables

**Table 1:**
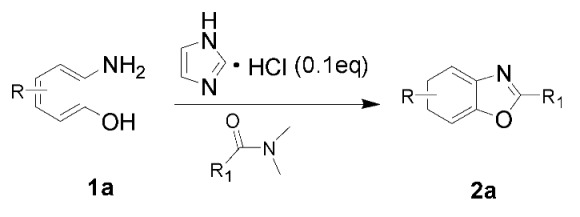
Synthesis of 2-substituted-benzoxazoles ^a^.

Entry	Substance		Product 1	Yield ^b^ (%)
	Substance 1	Substance 2		
1	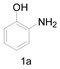	DMA^c^	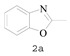	18
2	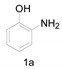	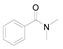	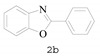	8
3	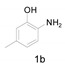	DMA	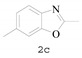	15
4	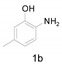	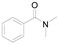	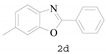	10

^a^*Conditions*: *O*-aminophenol derivatives (5.5 mmol, 1 equiv.), DMA (5 mL), *N*,*N*-dimethylbenzamide (11 mmol, 2 equiv.), imidazolium chloride (30 mol %), 140 °C, 6 h. ^b^ Isolated yields are given. ^C^*N*,*N*-dimethyl-acetamide.

**Table 2:**
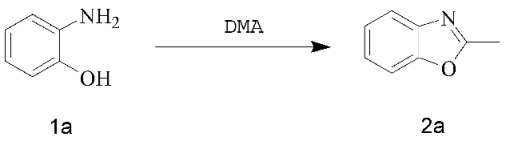
Optimization of reaction conditions ^a.^

Entry	Cat (equiv.)	Solvent	Temp	Time	Yield ^b^ (%)
1	-	DMA	140	8	-
2	HCl (0.5)	DMA	140	8	trace
3	Imidazolium chloride (0.3)	DMA	140	8	18
4	Imidazolium chloride (0.3)	DMA	150	8	43
5	Imidazolium chloride (0.3)	DMA	160	8	60
6	Imidazolium chloride (0.3)	DMA	170	8	62
7	Imidazolium chloride (0.3)	DMA	180	8	65
8	Imidazolium chloride (0.5)	DMA	160	8	87
9	Imidazolium chloride (0.8)	DMA	160	8	85
10	Imidazolium chloride (1.0)	DMA	160	8	88
11 ^c^	Imidazolium chloride (0.5)	Xylenes	140	10	15
12 ^c^	Imidazolium chloride (0.5)	H_2_O	100	10	Trace
13 ^c^	Imidazolium chloride (0.5)	Benzene	90	10	Trace

^a^ All reactions were carried out on an approximately 0.6 g scale using *O*-aminophenol (**1a**) (5.5 mmol, 1 equiv.), solvent (5 mL), imidazolium chloride (30–100 mol %). ^b^ Isolated yields are given. ^c^ DMA (0.54 mL, 6.6 mmol, 1.2 equiv.) and solvent (5 mL).

**Table 3:**
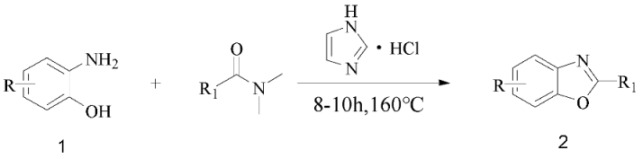
Substrate scope with respect to substituted 2-aminophenols ^a^.

Entry	Substance		Time (h)	Product	Yield ^b^ (%)
	Substance 1	Substance 2			
1	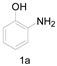	DMA	8	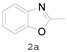	80
2 ^C^	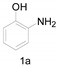	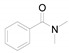	10	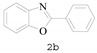	86
3 ^C^	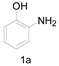	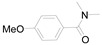	10	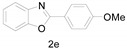	88
4 ^C^	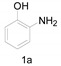	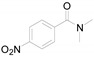	10	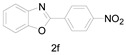	52
5	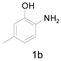	DMA	8	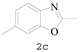	86
6	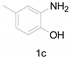	DMA	8	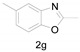	84
7 ^C^	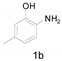	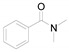	10	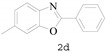	83
8	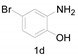	DMA	8	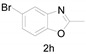	87
9 ^C^	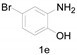	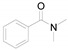	10	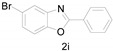	80

^a^ Experiments were performed with substance 1 (5 mmol) and DMA (5 mL) at 160 °C for 8 h. ^b^ Yields after column purification. ^C^ Experiments were performed with substance 1 (5 mmol) and DMA derivatives (10 mmol) at 160 °C for 10 h.

**Table 4:**
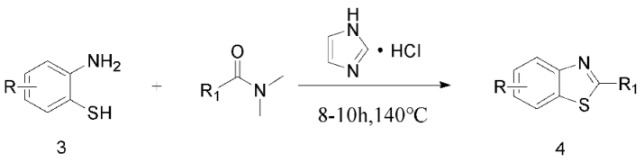
Substrate scope with respect to substituted 2-aminothiophenols ^a^.

Entry	Substance		Time (h)	Product	Yield ^b^ (%)
	Substance 1	Substance 2			
1	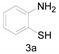	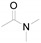	10	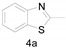	82
2	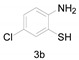	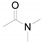	10	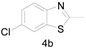	85
3	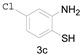	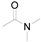	10	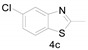	80
4	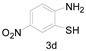	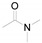	10	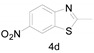	75
5	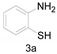	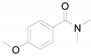	10	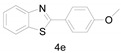	87
6	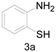	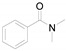	10	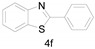	79
7	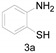	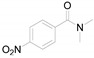	10	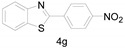	60
8	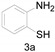	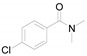	10	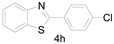	82
9	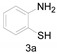	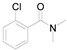	10	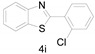	79
10	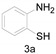	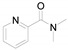	10	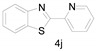	75
11	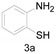	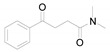	10	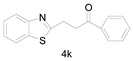	82

^a^ Experiments were performed with 2-amino-benzenethiol derivatives (5 mmol), DMA derivatives (10 mmol) and DMA (5 mL) at 140 °C for 8–10 h. ^b^ Yields after column purification.

## Supplementary Materials

The following are available online, general procedure for 2-substituted benzoxazoles and 2-substituted benzothiazoles of cyclization reaction and ^1^H-NMR and ^13^C-NMR spectra of all products.
