# Supplementary material for: Eco-Friendly Syntheses of 2-Substituted Benzoxazoles and 2-Substituted Benzothiazoles from 2-Aminophenols, 2-Aminothiophenols and DMF Derivatives in the Presence of Imidazolium Chloride
Source: Molecules. 2019 Jan 4;24(1):174. doi: 10.3390/molecules24010174 (PMC6337447; doi:10.3390/molecules24010174)
Supplement: Supplementary file 1 [file molecules-24-00174-s001.pdf]

Supporting Information

Eco-friendly syntheses of 2-substituted benzoxazoles and 2-substituted benzothiazoles from 2-aminophenols, 2-aminothiophenols and DMF derivatives in the presence of imidazolium chloride

Qingqiang Tian[1], Wen Luo[1], Zongjie Gan, Dan Li, Zeshu Dai, Huajun Wang, Xuetong Wang and Jianyong Yuan*

Department of Medicinal Chemistry, College of Pharmacy, Chongqing Medical University, Chongqing 400016, PR China

Tqingqiang@163.com (Q.T.); Lwen1129@163.com (W.L.); gzj@cqmu.edu.cn.(Z.G.); ld28115@163.com (D.L.) daizs11@163.com (Z.D.); Wanghuajun333@163.com (H.W.); wxy998298@163.com (X.W.).

*Corresponding authors: Jianyong Yuan

Email:enediyne@163.com Tel.:86-23-68485161

[1]These authors contributed equally to this work.

Contents

1. General information 2

2. General Procedures 2

2.1 General procedure for the synthesis of benzoxazole derivatives (**2a - 2d**)............... 2

2.2 General procedure for the synthesis of benzoxazole derivatives (**2a**, **2c**, **2g** and **2h**) and benzothiazole derivatives (**4a-4d**). 3

2.3 General procedure for the synthesis of benzoxazole derivatives (**2b**, **2d**, **2e**, **2f**, **2i**) and benzothiazole derivatives(**4e-4k**) 3

3. Characterization of products 2-substituted benzoxazoles and 2-substituted benzothiazoles.. 3

4. References 10

1. General information

All reagents were purchased from Ltd. (Shenzhen, China), Meyer Reagent Co., Ltd. (Shanghai, China), Macklin Reagent Co., Ltd. (Shanghai, China), Chongqing Chuandong Chemical Co., Ltd. (Chongqing, China). etc., and used without further purification. ^{1}H and ^{13}C NMR spectra were recorded on a Bruker Avancelll NMR spectrometer (600MHz) in $CDCl_3$ internally referenced to tetramethylsilane (TMS) or $CDCl_3$ signals. Chemical shifts are reported in ppm and coupling constants (J) in Hz. Chromatography was carried out on silica gel (200-300 mesh, Merck) using gravity flow. All substrates are known compounds according to the literature.[1] H and ^{13} C NMR spectra were recorded in $CDCl_3$ and DMSO-d6 on a Bruker Ascend-III 600 MHz and 600 MHz spectrometer using TMS as an internal standard. The residual solvent signals were used as references and the chemical shifts converted to the TMS scale ($CDCl_3$: δ H = 7.25-7.26 ppm, δ C = 77.23 ppm; DMSO-d 6 : δ H = 2.51 ppm, δ C = 39.51 ppm).

2. General Procedures

2.1 General procedure for the synthesis of benzoxazole derivatives (2a - 2d)

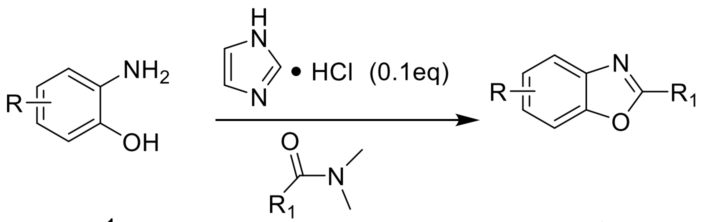

1a 2a

A mixture of **1a** (0.6g, 5.5 mmol, 1 equiv), imidazolium chloride (0.17g, 1.65mmol, 0.3equiv) and N,N-dimethylacetamide 5ml was stirred at 140°C for 8h. When the reaction was completed. Water (15ml) and ethyl acetate (20ml) were added with stirring to the reaction mixture. The organic layer was extracted and dried over anhydrous Na_2SO_4, filtered and concentrated under reduced pressure. The resulting residue was purified by column chromatography on silica gel using PE/EA as eluent to give the target product **2a**.

2.2 General procedure for the synthesis of benzoxazole derivatives (2a, 2c, 2g and 2h) and benzothiazole derivatives (4a-4d).

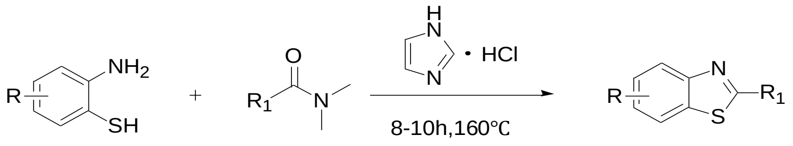

3a 4a

A tube-type schlenk flask was charged with **1a** (0.6g, 5.5 mmol, 1 equiv), imidazolium chloride (0.28g, 1.65 mmol, 0.5equiv) and N,N-dimethylacetamide 5ml was stirred at 160°C for 8h. When the reaction was completed. Water (15ml) and ethyl acetate (20ml) were added with stirring to the reaction mixture. The organic layer was extracted and dried over anhydrous Na_2SO_4, filtered and concentrated under reduced pressure. The resulting residue was purified by column chromatography on silica gel using PE/EA as eluent to give the corresponding product **2a**.

2.3 General procedure for the synthesis of benzoxazole derivatives (2b, 2d, 2e, 2f, 2i) and benzothiazole derivatives (4e-4k)

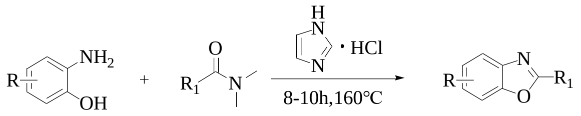

1a **2a**

To a mixture of **1a** (0.6g, 4.8 mmol, 1equiv), imidazolium chloride (0.25g, 2.4 mmol, 0.5equiv) and N,N-dimethylbenzamide (1.43g, 9.6 mmol, 2 equiv) was added. The mixture was stirred at 160°C for 10h. after completion of the reaction.15ml water was added and the resulting mixture was extracted with 20ml EA thrice, and the combined organic layers were dried over anhydrous Na_2SO_4 and concentrated. The residue was purified by column chromatography on silica gel using PE/EA as eluent to obtain the pure desired product.

3. Characterization of products 2-substituted benzoxazoles and 2-substituted benzothiazoles.

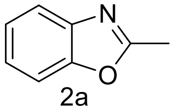

2a

2-methylbenzo[d]oxazole (2a)[1]: The product was obtained as yellow liquid in 80% yield. ^{1}H NMR (600 MHz, $CDCl_3$) δ 7.64 (d, J = 7.2 Hz, 1H), 7.45 (d, J = 7.6 Hz, 1H), 7.29 – 7.25 (m, 2H), 2.61 (s, 3H). ^{13}C NMR (151 MHz, $CDCl_3$) δ 163.74, 150.94, 141.49, 124.39, 124.04, 119.38, 110.15, 14.46.

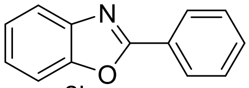

2b

2-phenylbenzo[d]oxazole (2b)[2]: The product was obtained as white soild in 86% yield. MP:101-103°C. ^{1}H NMR (600 MHz, $CDCl_3$) δ 8.27 (d, J = 6.3 Hz, 2H), 7.80 – 7.78 (m, 1H), 7.60 – 7.58 (m, 1H), 7.53 (d, J = 7.0 Hz, 3H), 7.36 (dd, J = 6.0, 3.1 Hz, 2H). ^{13}C NMR (151 MHz, $CDCl_3$) δ 162.02, 149.72, 140.96, 130.55, 127.90, 126.63, 126.07, 124.12, 123.59, 118.97, 109.58.

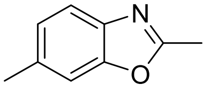

2c

2, 6-dimethylbenzo[d]oxazole(2c)[3]: The product was obtained as yellow liquid in 86% yield. ^{1}H NMR (600 MHz, $CDCl_3$) δ 7.42 (d, J = 8.1 Hz, 1H), 7.17 (s, 1H), 7.01 (d, J = 8.0 Hz, 1H), 2.51 (s, 3H), 2.37 (s, 3H). ^{13}C NMR (151 MHz, $CDCl_3$) δ 163.23, 151.26, 139.25, 134.71, 125.22, 118.70, 110.39, 21.63, 14.46.

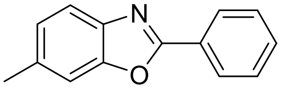

2d

6-methyl-2-phenylbenzo[d]oxazole(2d)[4]: The product was obtained as yellow solid in 83% yield. MP: 90-92°C. ^{1}H NMR (600 MHz, $CDCl_3$) δ 8.17 – 8.15 (m, 2H), 7.57 (d, J = 8.1 Hz, 1H), 7.44 (d, J = 1.6 Hz, 3H), 7.31 (s, 1H), 7.10 (d, J = 8.1 Hz, 1H), 2.43 (s, 3H). ^{13}C NMR (151 MHz, $CDCl_3$) δ 161.55, 150.03, 138.87, 134.56, 130.26, 127.85, 126.44, 126.32, 124.79, 118.31, 109.74, 20.78.

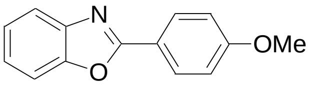

2e

2-(4-methoxyphenyl)benzo[d]oxazole（2e）[16]**:** The product was obtained as white solid in 88% yield. MP:97-100. ^{1}H NMR (600 MHz, $CDCl_3$) δ 8.20 (d, J = 8.5 Hz, 2H), 7.74 (d, J = 8.7 Hz, 1H), 7.56 (d, J = 6.8 Hz, 1H), 7.32 (h, J = 6.7, 6.1 Hz, 2H), 7.03 (d, J = 8.3 Hz, 2H), 3.89 (s, 3H).^{13}C NMR (151 MHz, $CDCl_3$) δ 163.17, 162.34, 150.66, 142.21, 129.42, 124.61, 124.43, 119.63, 114.37, 110.39, 55.47.

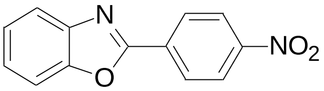

2f

2-(4-nitrophenyl)benzo[d]oxazole(2f)[5]: The product was obtained as yellow solid in 52% yield. MP: 166-167°C. ^{1}H NMR (600 MHz, $CDCl_3$) δ 7.74 (d, J = 6.9 Hz, 2H), 7.52 (t, J = 7.5 Hz, 2H), 7.42 (t, J = 7.8 Hz, 2H), 7.30 – 7.26 (m, 2H). ^{13}C NMR (151 MHz, $CDCl_3$) δ 160.67, 151.04, 149.42, 141.90, 132.80, 128.42, 126.37, 125.25, 124.25, 120.70, 110.96.

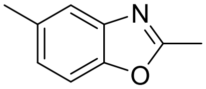

2g

2, 5-dimethylbenzo[d]oxazole(2g)[6]: The product was obtained as yellow liquid in 84% yield. ^{1}H NMR (600 MHz, $CDCl_3$) δ 7.42 (s, 1H), 7.30 (d, J = 22.9 Hz, 1H), 7.07 (d, J = 7.6 Hz, 1H), 2.60 (s, 3H), 2.44 (s, 3H). ^{13}C NMR (151 MHz, $CDCl_3$) δ 163.85, 149.19, 141.68, 133.82, 125.43, 119.34, 109.51, 21.38, 14.49.

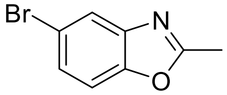

2h

5-bromo-2-methylbenzo[d]oxazole(2h)[7]: The product was obtained as yellow liquid in 87% yield. ^{1}H NMR (600 MHz, $CDCl_3$) δ 7.78 (d, J = 1.9 Hz, 1H), 7.39 (d, J = 8.6 Hz, 1H), 7.33 (d, J = 8.5 Hz, 1H), 2.64 (s, 3H). ^{13}C NMR (151 MHz, $CDCl_3$) δ 165.10, 149.94, 143.11, 127.45, 122.46, 116.83, 111.42, 14.56.

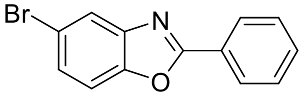

2i

5-bromo-2-phenylbenzo[d]oxazole(2i)[8]: The product was obtained as white solid in 80% yield. MP:108-110 °C. ^{1}H NMR (600 MHz, $CDCl_3$) δ 8.24 (d, J = 7.5 Hz, 2H), 7.91 (s, 1H), 7.58 – 7.52 (m, 3H), 7.46 (s, 2H). ^{13}C NMR (151 MHz, $CDCl_3$) δ 164.16, 149.74, 143.64, 131.97, 128.99, 128.10, 127.78, 126.60, 122.96, 117.33, 111.81.

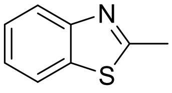

4a

2-methylbenzo[d]thiazole(4a)[9]: The product was obtained as yellow liquid in 82% yield. ^{1}H NMR (600 MHz, $CDCl_3$) δ 7.95 (d, J = 8.0 Hz, 1H), 7.80

(d, J = 7.3 Hz,1H), 7.43 (t, J = 7.7 Hz, 1H), 7.34 – 7.31 (m, 1H), 2.81 (s,3H). ^{13}C NMR (151 MHz, $CDCl_3$) δ 166.90, 153.36, 135.64, 125.90, 124.68, 122.37, 121.38, 20.09.

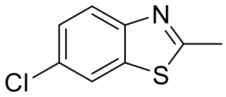

4b

6-chloro-2-methylbenzo[d]thiazole(4b)[10]: The product was obtained as yellow solid in 85% yield. MP:79-82 °C. ^{1}H NMR (600 MHz, $CDCl_3$) δ 7.80 (d, J = 8.6 Hz, 1H), 7.74 (s, 1H), 7.35 (d, J = 8.6 Hz, 1H), 2.78 (s, 3H). ^{13}C NMR (151 MHz, $CDCl_3$) δ 167.78, 151.49, 136.64, 130.79, 126.85, 123.05, 121.08, 20.13.

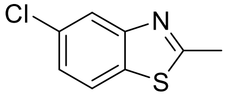

4c

5-chloro-2-methylbenzo[d]thiazole(4c)[1]: The product was obtained as white solid in 80% yield. MP:60-62 °C. ^{1}H NMR (600 MHz, $CDCl_3$) δ 7.93 (d, J = 2.0 Hz, 1H), 7.73 (d, J = 8.5 Hz, 1H), 7.33 (d, J = 6.5 Hz, 1H), 2.84 (s, 3H). ^{13}C NMR (151 MHz, $CDCl_3$) δ 169.04, 154.13, 133.85, 132.00, 125.24, 122.29, 122.09, 20.23.

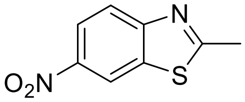

4d

2-methyl-6-nitrobenzo[d]thiazole(4d)[11]: The product was obtained as yellow solid in 75% yield. MP:161-162 °C. ^{1}H NMR (600 MHz, $CDCl_3$) δ 8.78 (s, 1H), 8.34 (d, J = 6.7 Hz, 1H), 8.04 (d, J = 8.9 Hz,1H), 2.93 (s, 3H).

^{13}C NMR (151 MHz, $CDCl_3$) δ 173.30, 157.09, 144.81, 136.02, 122.65, 121.59, 118.01, 20.71.

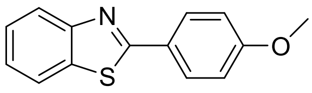

4e

2-(4-methoxyphenyl)benzo[d]thiazole(4e)[12] : The product was obtained as white solid in 87% yield. MP:119-120 °C. ^{1}H NMR (600 MHz, $CDCl_3$) δ 8.04 (d, J = 8.6 Hz, 3H), 7.87 (d, J = 7.6 Hz, 1H), 7.47 (t, J = 7.4 Hz, 1H), 7.35 (t, J = 7.6 Hz, 1H), 7.00 (d, J = 8.8 Hz, 2H), 3.87 (s, 3H). ^{13}C NMR (151 MHz, $CDCl_3$) δ 167.91, 161.96, 154.11, 134.80, 129.15, 126.36, 126.25, 124.83, 122.80, 121.53, 114.57, 114.39, 77.26, 77.13, 55.49.

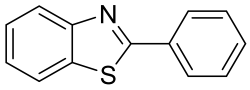

4f

2-phenylbenzo[d]thiazole (4f)[12]: The product was obtained as white solid in 79% yield. MP:111-113 °C ^{1}H NMR (600 MHz, $CDCl_3$) δ 8.12 – 8.08 (m, 3H), 7.91 (d, J = 8.0 Hz, 1H), 7.50 (p, J = 4.1 Hz, 4H), 7.39 (t, J = 7.6 Hz, 1H). ^{13}C NMR (151 MHz, $CDCl_3$) δ 168.15, 153.99, 135.00, 133.52, 131.06, 129.07, 127.62, 126.39, 125.26, 123.23, 121.66.

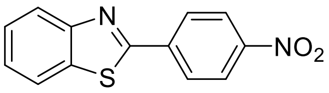

4g

2-(4-nitrophenyl)benzo[d]thiazole(4g) [13] : The product was obtained as yellow solid in 60% yield. MP: 224-226°C. ^{1}H NMR (600 MHz, $CDCl_3$) δ 8.29 (d, J = 8.9 Hz, 2H), 8.21 (d, J = 8.8 Hz, 2H), 8.07 (d, J = 8.2 Hz, 1H), 7.89 (d, J = 7.8 Hz, 1H), 7.51 – 7.48 (m, 1H), 7.42 – 7.39 (m,1H). ^{13}C NMR

(151 MHz, $CDCl_3$) δ 163.82, 153.08, 148.02, 138.16, 134.46, 127.23, 125.90, 125.21, 123.30, 122.92, 120.82.

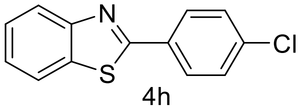

4h

2-(4-chlorophenyl)benzo[d]thiazole(4h)[14]: The product was obtained as white solid in 82% yield. MP:114-115 °C. ^1H NMR (600 MHz, $CDCl_3$) δ 8.07 (d, J = 8.2 Hz, 1H), 8.04 – 8.01 (m, 2H), 7.90 (dd, J = 8.0, 1.1 Hz, 1H), 7.50 (ddd, J = 8.2, 7.1, 1.2 Hz, 1H), 7.47 – 7.45 (m, 2H), 7.41 – 7.38 (m, 1H). ^{13}C NMR (151 MHz, $CDCl_3$) δ 166.65, 154.02, 137.06, 135.04, 132.08, 129.29, 128.73, 126.52, 125.45, 123.30, 121.67.

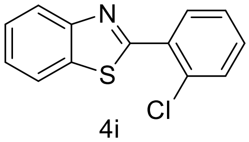

4i

2-(2-chlorophenyl)benzo[d]thiazole(4i)[2]: The product was obtained as white solid in 79% yield. MP: 81-83 °C. ^1H NMR (600 MHz, $CDCl_3$) δ 8.24 – 8.19 (m, 1H), 8.16 – 8.12 (m, 1H), 7.95 (dd, J = 8.1, 1.1 Hz, 1H), 7.57 – 7.51 (m, 2H), 7.47 – 7.39 (m, 3H). ^{13}C NMR (151 MHz, $CDCl_3$) δ 164.20, 152.38, 136.05, 132.71, 132.19, 131.76, 131.17, 130.81, 127.12, 126.32, 125.47, 123.43, 121.40.

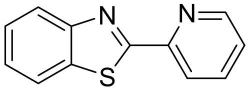

4j

2-(pyridin-2-yl)benzo[d]thiazole(4j)[15]: The product was obtained as white solid in 75% yield. MP:133-135 °C. ^1H NMR (600 MHz, $CDCl_3$) δ 8.70 (d, J = 4.0 Hz, 1H), 8.39 (d, J = 8.0 Hz, 1H), 8.10 (d, J = 8.2 Hz, 1H), 7.97 (d, J =

7.6 Hz, 1H), 7.86 (t, J = 7.7 Hz, 1H), 7.51 (t, J = 7.0 Hz, 1H), 7.44 – 7.38 (m, 2H).^{13}C NMR (151 MHz, $CDCl_3$) δ 169.35, 154.22, 151.37, 149.65, 137.05, 136.11, 126.30, 125.67, 125.29, 123.57, 122.02, 120.81.

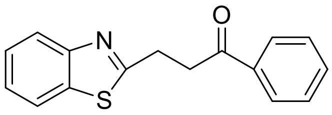

4k

3-Benzothiazol-2-yl-1-phenyl-propan-1-one (4k) [17]: The product was obtained as white solid in 82% yield. MP: 93-95 °C. ^{1}H NMR (600 MHz, $CDCl_3$) δ 8.02 (d, J = 7.8 Hz, 2H), 7.95 (d, J = 8.1 Hz, 1H), 7.83 (d, J = 8.0 Hz, 1H), 7.57 (t, J = 7.4 Hz, 1H), 7.50 – 7.40 (m, 3H), 7.34 (t, J = 7.6 Hz, 1H), 3.66 (t, J = 7.1 Hz, 2H), 3.57 (t, J = 7.1 Hz, 2H). ^{13}C NMR (151 MHz, $CDCl_3$) δ 197.87, 170.59, 153.09, 136.50, 135.24, 133.36, 128.69, 128.14, 125.97, 124.85, 122.52, 121.54, 37.64, 28.29.

4. References

1 Mayo, M. S. ; Yu, X. ; Zhou, X. ; Feng, X. ; Yamamoto, Y. ; Bao, M. convenient synthesis of benzothiazoles and benzimidazoles through broensted acid catalyzed cyclization of 2-amino thiophenols/anilines with β-diketones. *Org. Lett*. **2014**, 45 764-767.

2 Bochatay, V. N. ; Boissarie, P. J. ; Murphy, J. A. ; Suckling, C. J. ; Lang, S. Mechanistic exploration of the palladium-catalyzed process for the synthesis of benzoxazoles and benzothiazoles. *J.Org. Chemy*. **2013**, 78, 1471-1477.

3 Rancan, E. ; Fabio Aricò ; Quartarone, G. ; Ronchin, L. ; Vavasori, A. Acid catalyzed direct-amidation–dehydrocyclization of 2-hydroxy-acetophenones to benzoxazoles by a one-pot sustainable synthesis. *Catal. Letters*. **2015**, 145, 939-946.

4 Wang, Y. ; Wu, C. ; Nie, S. ; Xu, D. ; Yu, M. ; Yao, X. ligand-promoted, copper nanoparticles catalyzed one-pot synthesis of substituted benzoxazoles from 2-bromoanilines and acyl chlorides. *Tetrahedron Lett*. **2016**, 47, 6827-6832.

5 Azizian, J. ; Torabi, P. ; Noei, J. Synthesis of benzimidazoles and benzoxazoles using TiCl3OTf in ethanol at room temperature. *Tetrahedron Lett*. **2016**, 57, 185-188.

6 Aksenov, N. A. ; Aksenov, A. V. ; Nadein, O. N. ; Aksenov, D. A. ; Smirnov, A. N. ; Rubin, M. One-pot synthesis of benzoxazoles via the metal-free ortho-C-H functionalization of phenols with nitroalkanes. *Rsc Adv*. **2015**, 5, 71620-71626.

7 Zhang, X. ; Huang, R. ; Jérôme Marrot ; Coeffard, V. ; Xiong, Y. Hypervalent iodine-mediated synthesis of benzoxazoles andbenzimidazoles via an oxidative rearrangement. *Tetrahedron*, **2015**, 71, 700-708.

8 Wang, L. ; Ma, Z. G. ; Wei, X. J. ; Meng, Q. Y. ; Yang, D. T. ; Du, S. F. Synthesis of 2-substituted pyrimidines and benzoxazoles via a visible-light-driven organocatalytic aerobic oxidation: enhancement of the reaction rate and selectivity by a base. *Green Chem*, **2014**,16, 3752-3757.

9 Mortimer, C. G. ; Wells, G. ; Crochard, J. P. ; Stone, E. L. ; Bradshaw, T. D. ; Stevens, M. F. G.; Antitumor benzothiazoles. 26.1 2-(3,4-dimethoxyphenyl)-5-fluorobenzothiazole (gw 610, nsc 721648), a simple fluorinated 2-arylbenzothiazole, shows potent and selective inhibitory activity against lung, colon, and breast cancer cell lines. *J. Med. Chem*, **2006**,49, 179-185.

10 Huang, X. ; Tang, J. Solid phase synthesis of benzothiazole and thiophene derivatives based on resin-bound cyclic malonic acid ester. *Tetrahedron*. **2003**, 59, 4851-4856.

11 T. S. ; Yamamoto, Y. Carbon−carbon bond cleavage of diynes through the hydroamination with transition metal catalysts.*JACS*, **2003**, 125, 6646-6647.

12 Sung, G. H. ; Lee, I. H. ; Bo, R. K. ; Shin, D. S. ; Kim, J. J. ; Lee, S. G., Yong, J. Y. Eco-friendly atom-economical synthesis of 2-substituted-benzo[d]thiazoles and2-substituted-benzo[d]oxazoles using 2-acylpyridazin-3(2h)-ones. *Tetrahedron*. **2013**, 69, 3530-3535.

13 Ranjit S , Liu X . Direct Arylation of Benzothiazoles and Benzoxazoles with Aryl Boronic Acids. *Eur. J Chem.*.**2011**, 17, 1105 – 1108.

14 Kumar, P. ; Meenakshi ; Kumar, S. ; Kumar, A. ; Hussain, K. ; Kumar, S. Solvent-free one pot synthesis of 2-aryl/heteroaryl benzothiazoles using hypervalent iodine (III) reagents. *J.Heterocycl. Chem*. **2012**, 49, 1243-1249.

15 Jiang, H. ; Huang, Y. ; Yan, D. ; Wang, X. ; Zhou, P. ; Wu, W. Controllably, carbon-carbon triple bond as one-carbon synthon to assembly of benzothiazole framework. *Chem. Commun*. **2018**, 54, 1742-1745.

16 Teo Y C, Riduan S N, Zhang Y. Iodine-mediated arylation of benzoxazoles with aldehydes. *Green Chem*. **2013**, 15, 2365-2368.

17 Lu, S. C.; Li, H. S.; Xu, S.; Duan, G. Y. silver catalyzed C2-selective direct alkylation of Heteroarenes with Tertiary Cycloalkanols. *Org. Biomol. Chem*. **2017**, 15, 324-327.

5. ^{1}H NMR and ^{13}C NMR spectra of

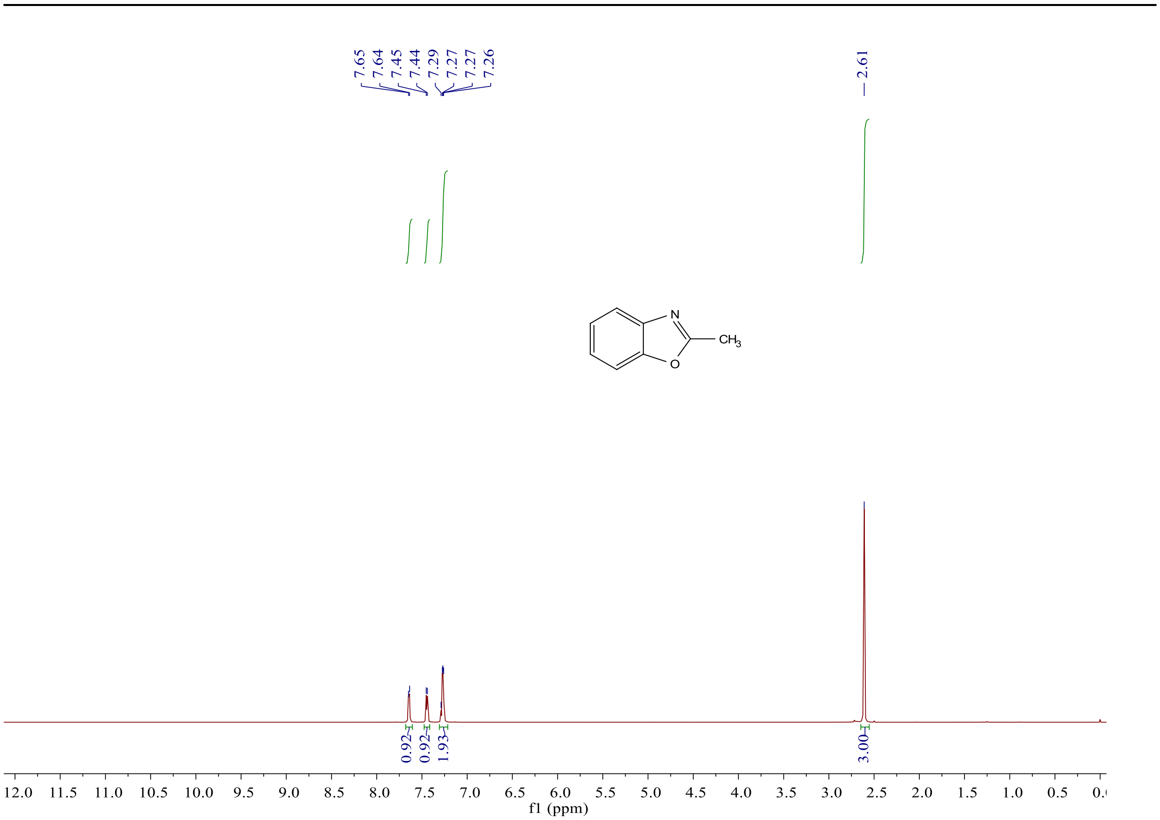

Figure1 ^{1}H NMR spectra of 2-methylbenzo[d]oxazole (**2a**) (solvent $CDCl_3$)

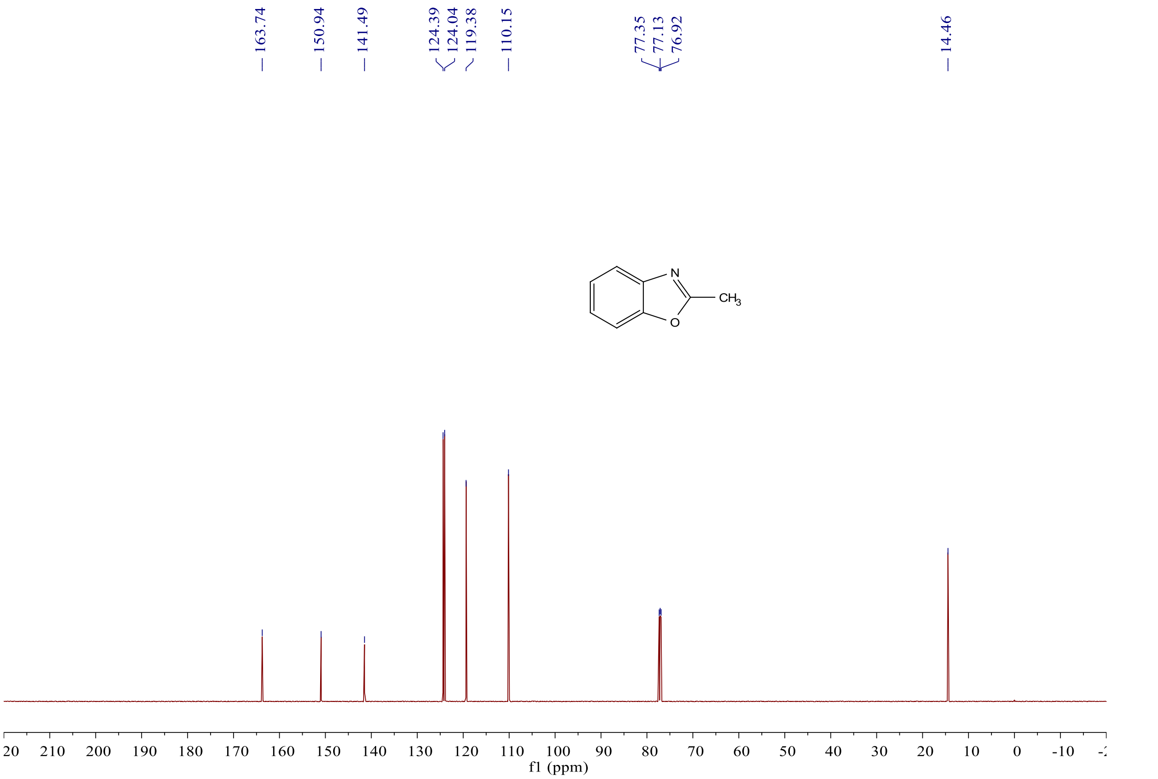

Figure2 ^{13}C NMR spectra of 2-methylbenzo[d]oxazole (**2a**) (solvent $CDCl_3$)

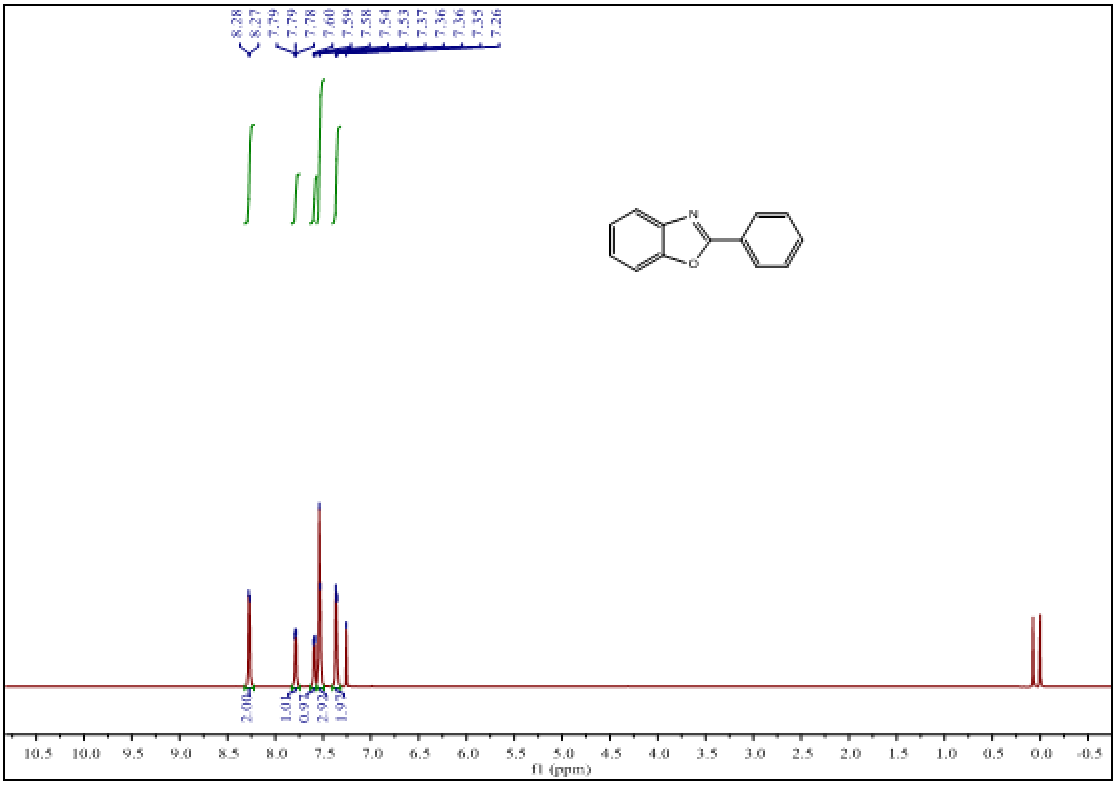

Figure3 ^{1}H NMR spectra of 2-phenylbenzo[d]oxazole (**2b**) (solvent $CDCl_3$)

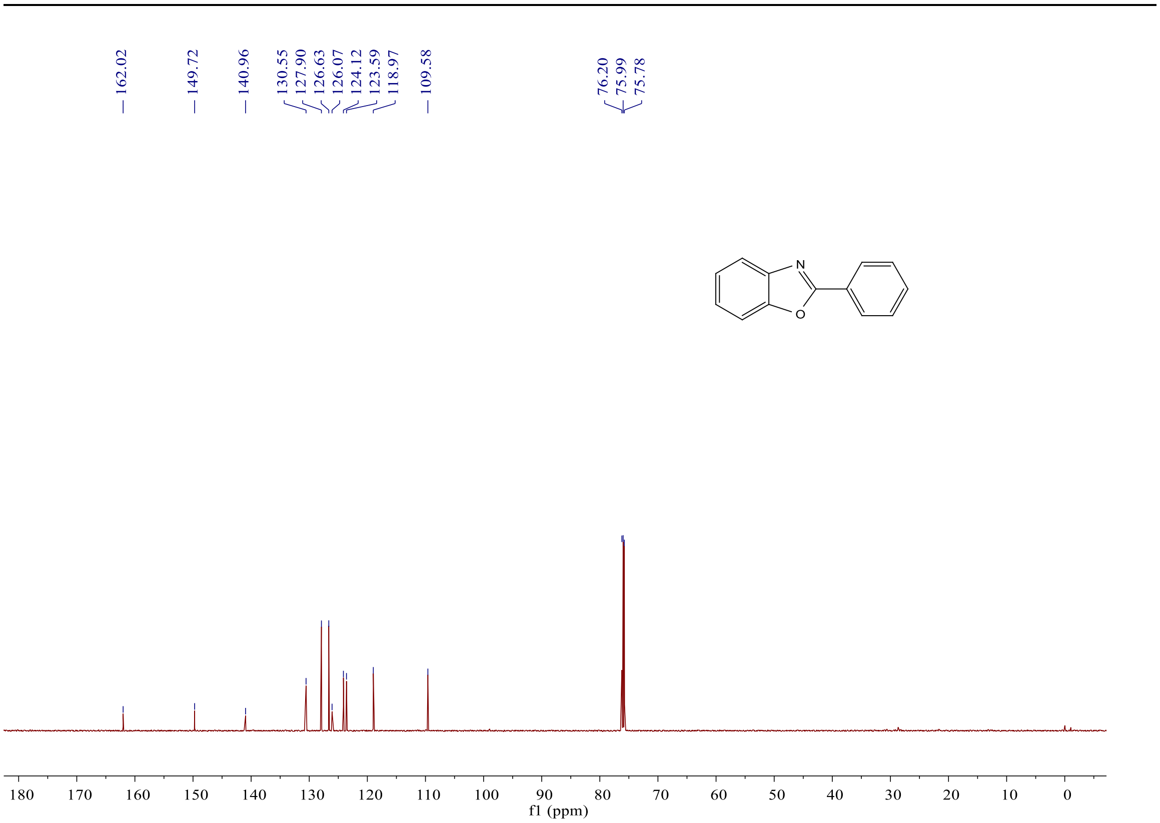

Figure4 ^{13}C NMR spectra of 2-phenylbenzo[d]oxazole (**2b**) (solvent $CDCl_3$)

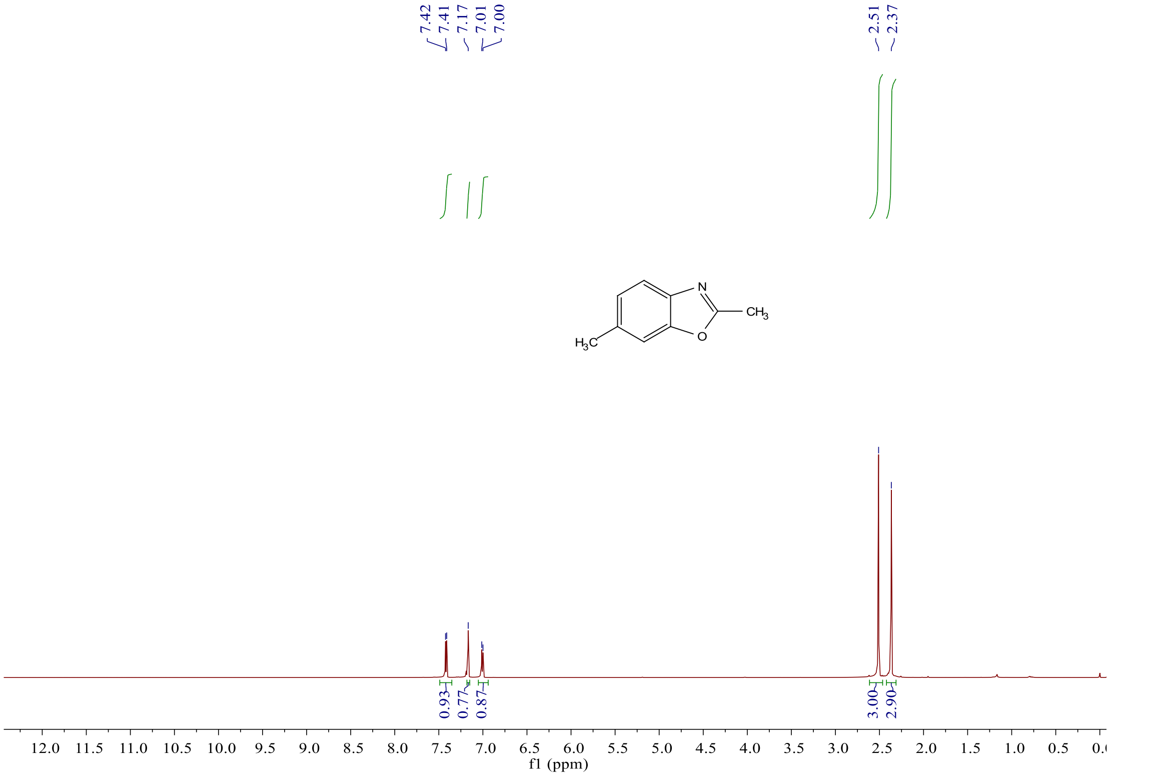

Figure5 ^{1}H NMR spectra of 2, 6-dimethylbenzo[d]oxazole(**2c**) (solvent $CDCl_3$)

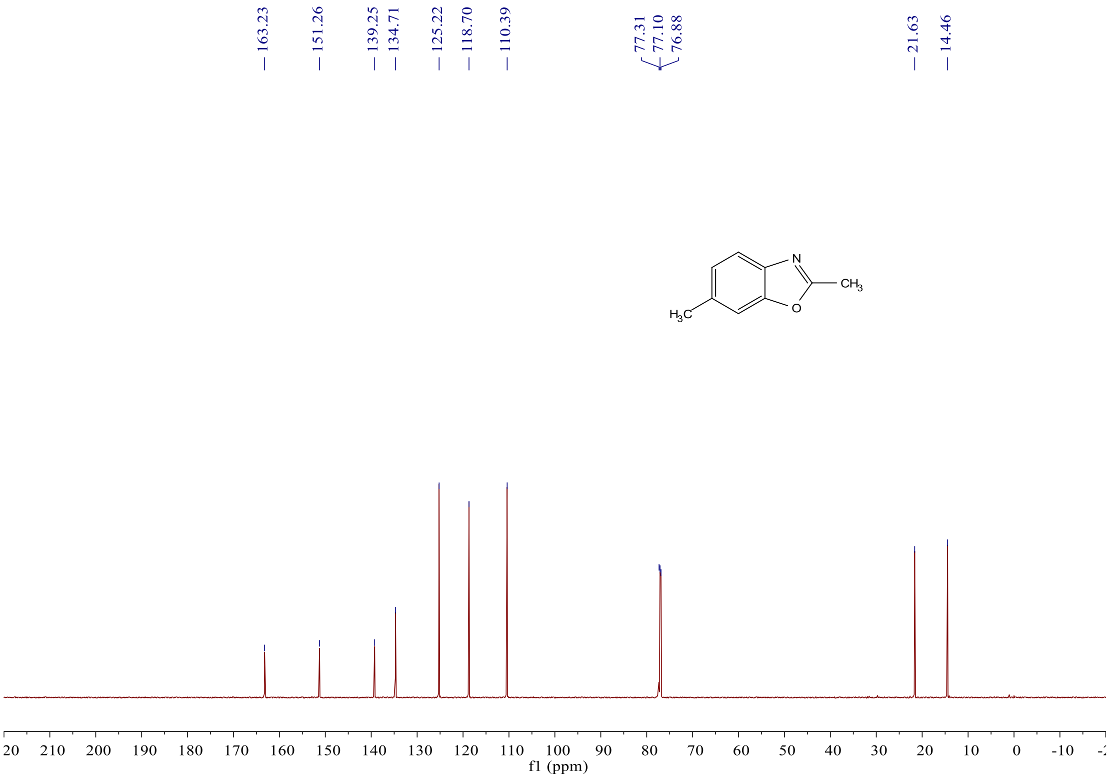

Figure6 ^{13}C NMR spectra of 2, 6-dimethylbenzo[d]oxazole(**2c**) (solvent $CDCl_3$)

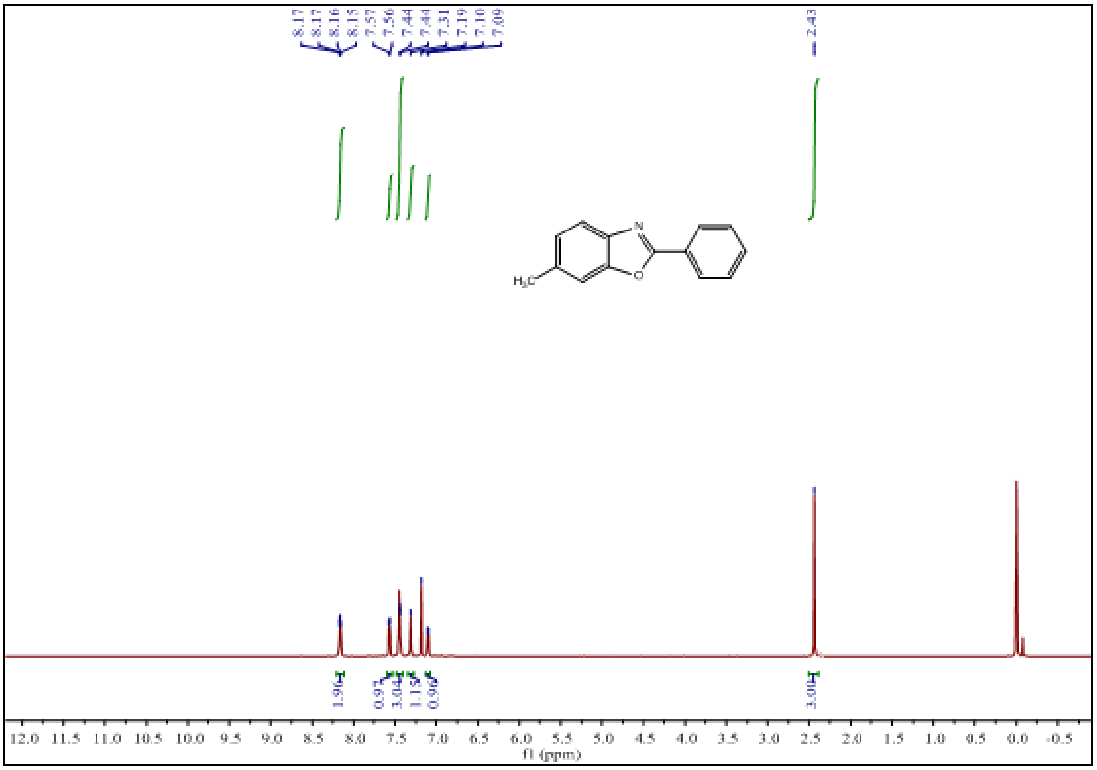

Figure7 ^{1}H NMR spectra of 6-methyl-2-phenylbenzo[d]oxazole(**2d**) (solvent $CDCl_3$)

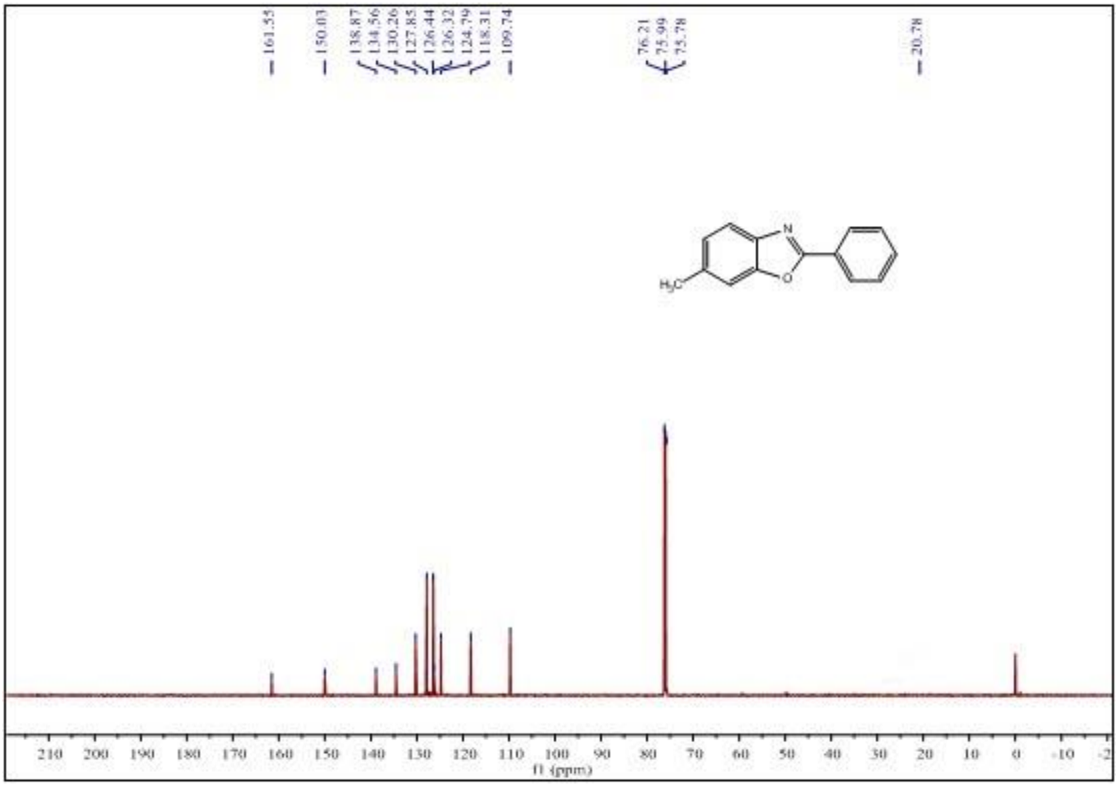

Figure8 ^{13}C NMR spectra of 6-methyl-2-phenylbenzo[d]oxazole(**2d**) (solvent $CDCl_3$)

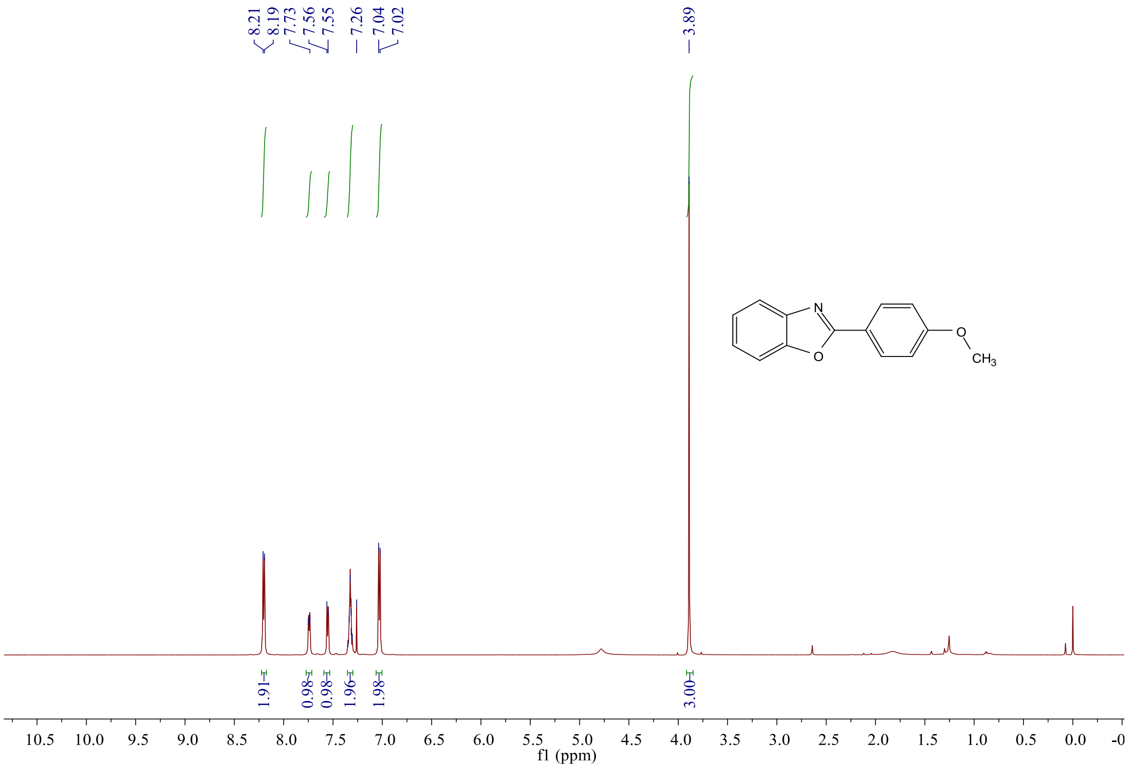

Figure9 ^{1}H NMR spectra of 2-(4-methoxyphenyl)benzo[d]oxazole（2e）(solvent $CDCl_3$)

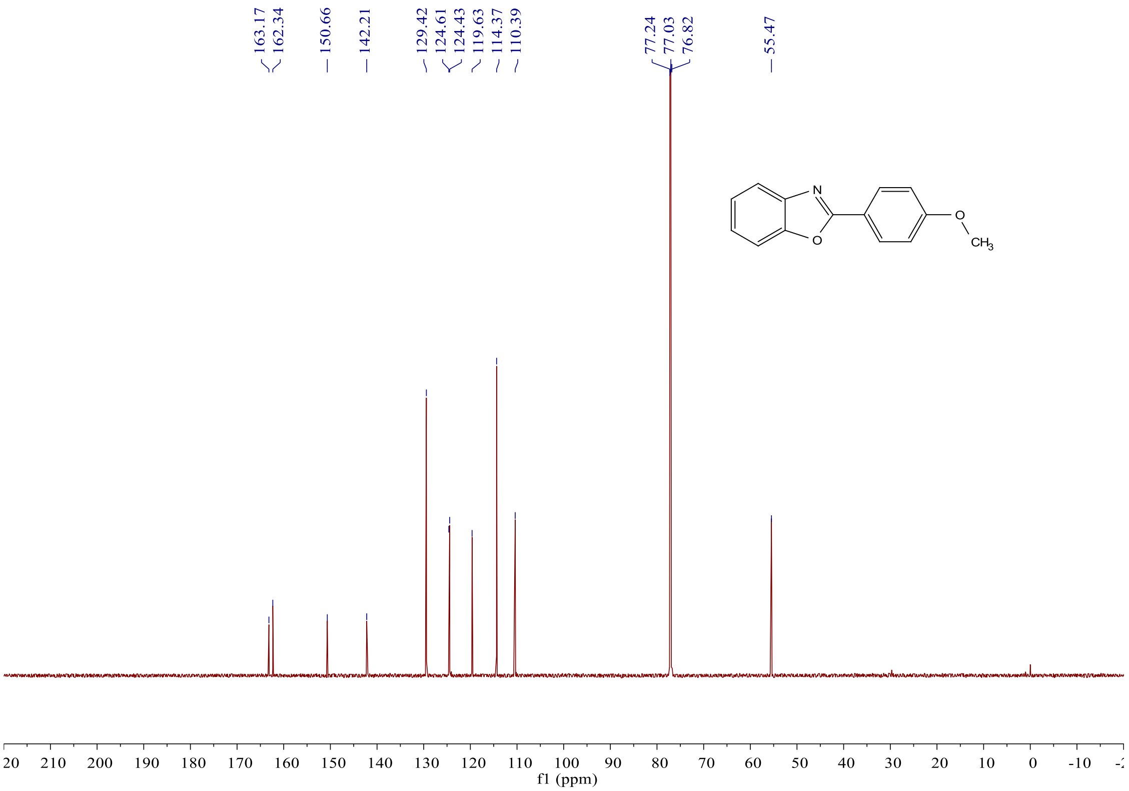

Figure10 ^{13}C NMR spectra of 2-(4-methoxyphenyl)benzo[d]oxazole（2e）(solvent $CDCl_3$)

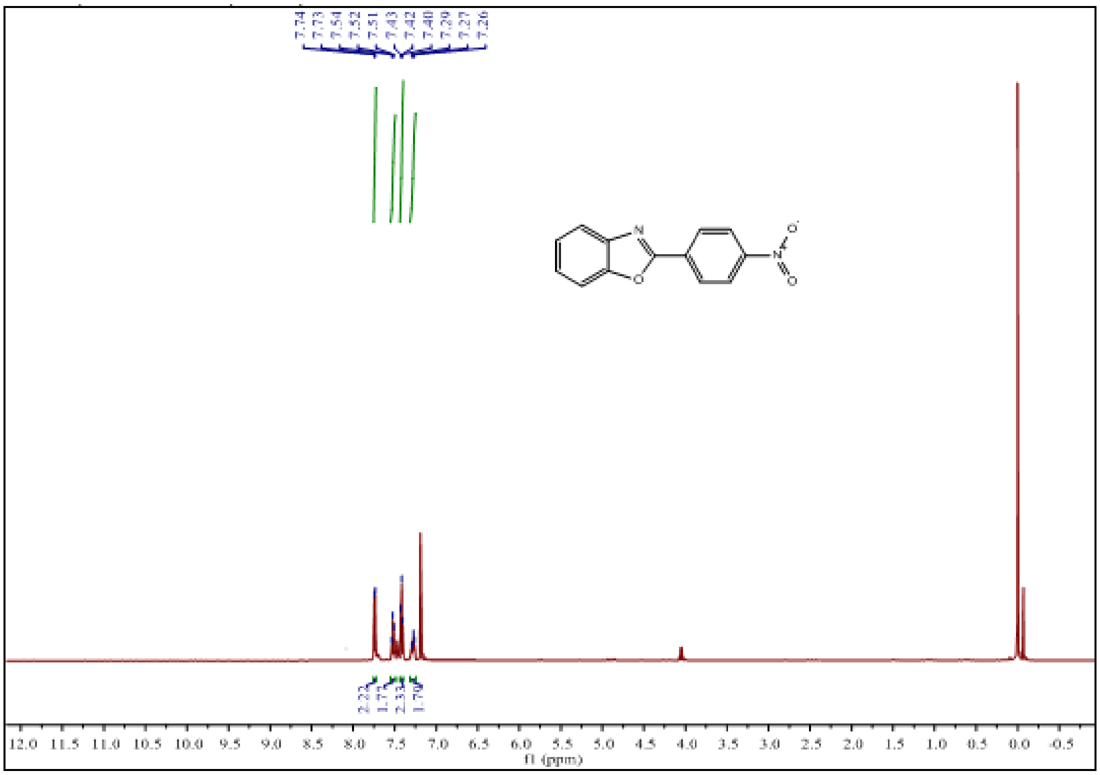

Figure11 ^{1}H NMR spectra of 2-(4-nitrophenyl)benzo[d]oxazole(**2f**) (solvent $CDCl_3$)

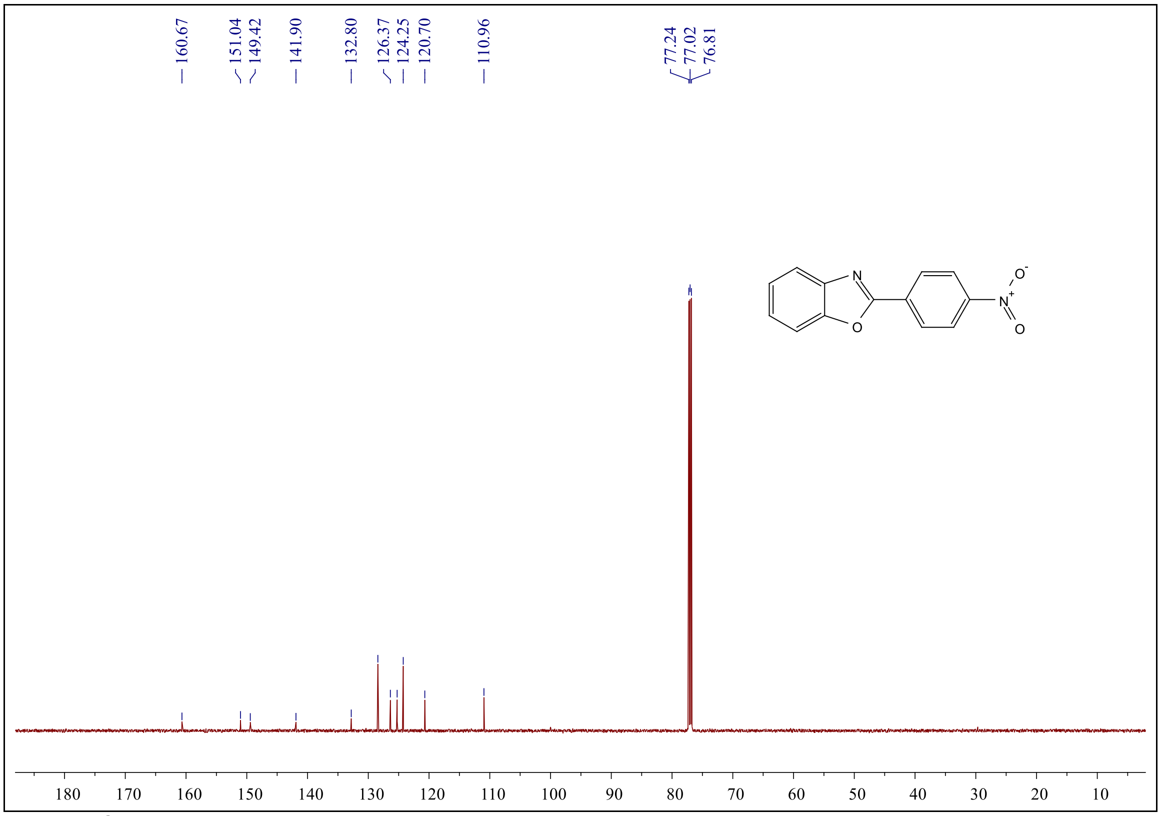

Figure12 ^{13}C NMR spectra of 2-(4-nitrophenyl)benzo[d]oxazole(**2f**) (solvent $CDCl_3$)

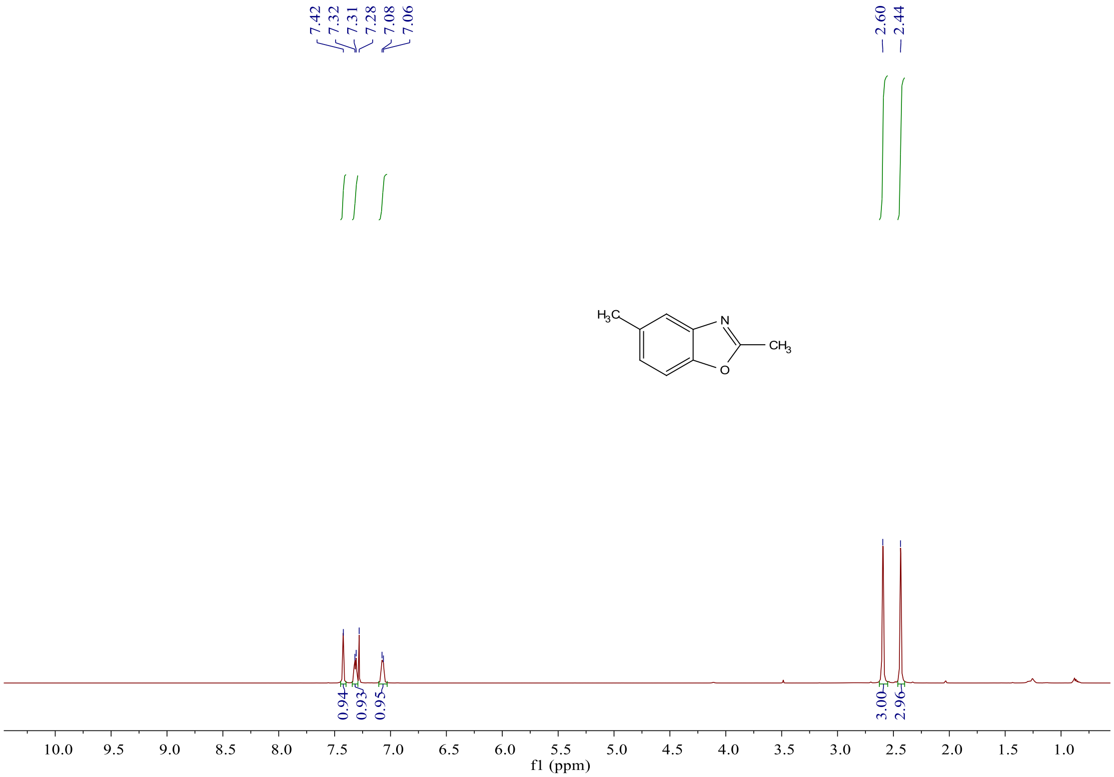

Figure13 ^{1}H NMR spectra of 2, 5-dimethylbenzo[d]oxazole(**2g**) (solvent $CDCl_3$)

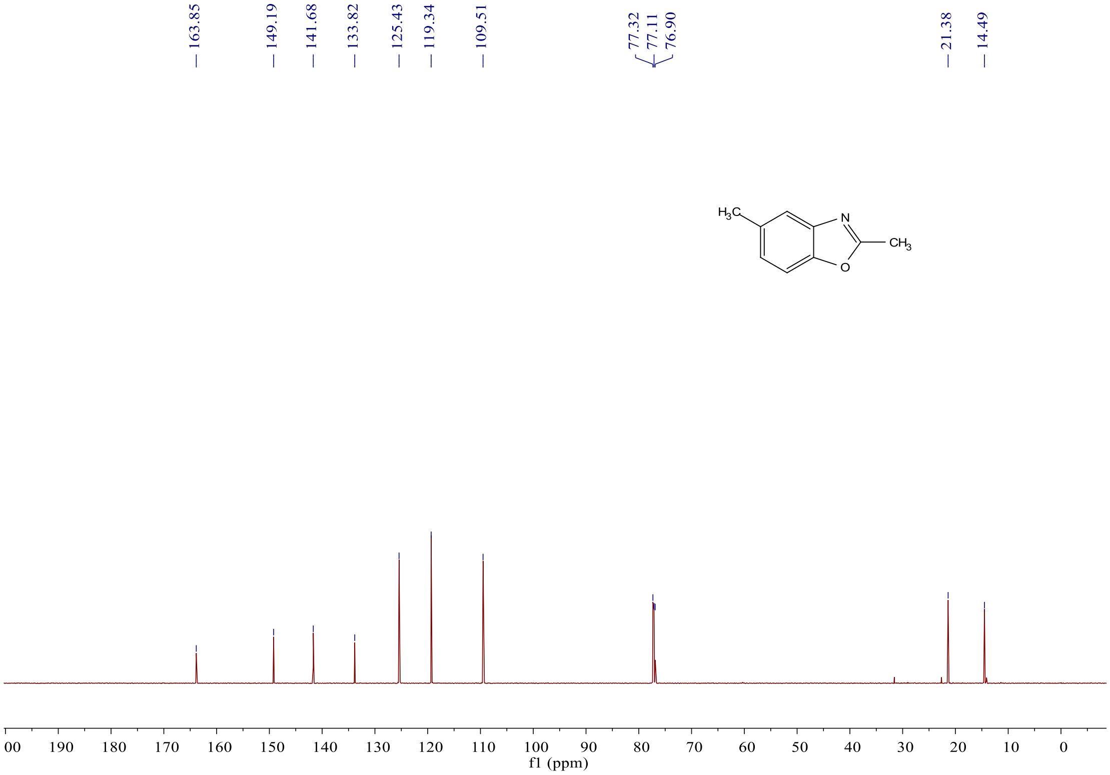

Figure14 ^{13}C NMR spectra of 2, 5-dimethylbenzo[d]oxazole(**2g**) (solvent $CDCl_3$)

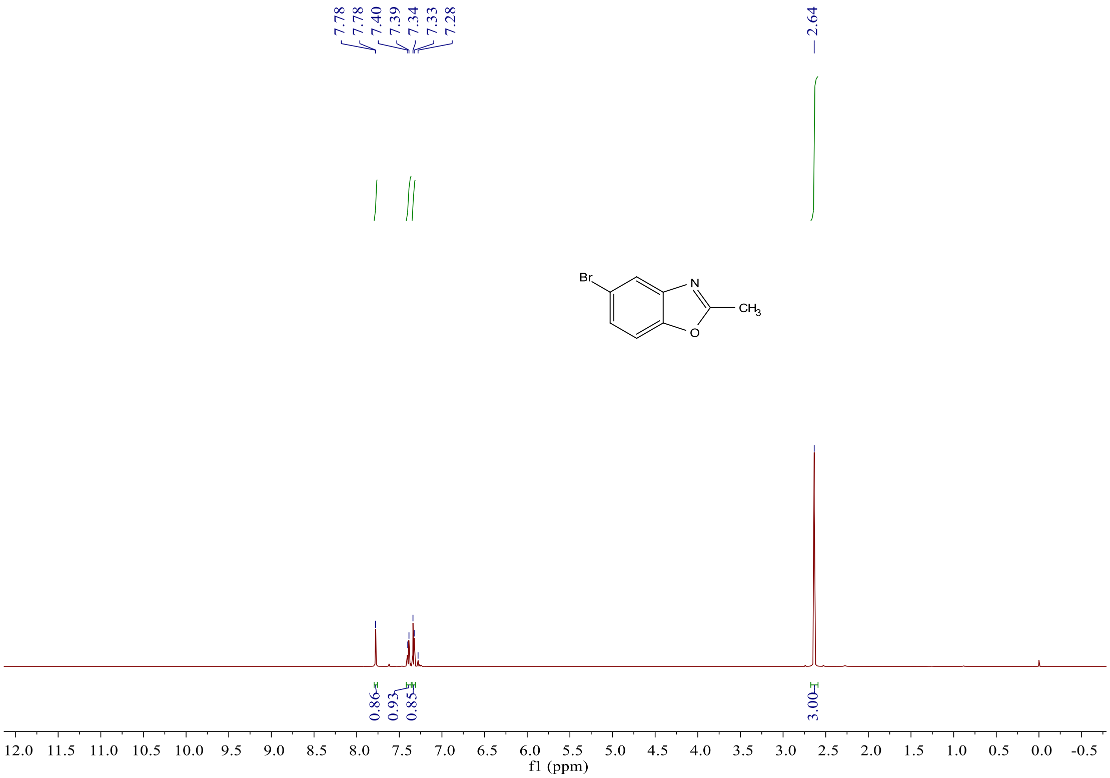

Figure15 ^{1}H NMR spectra of 5-bromo-2-methylbenzo[d]oxazole(**2h**) (solvent $CDCl_3$)

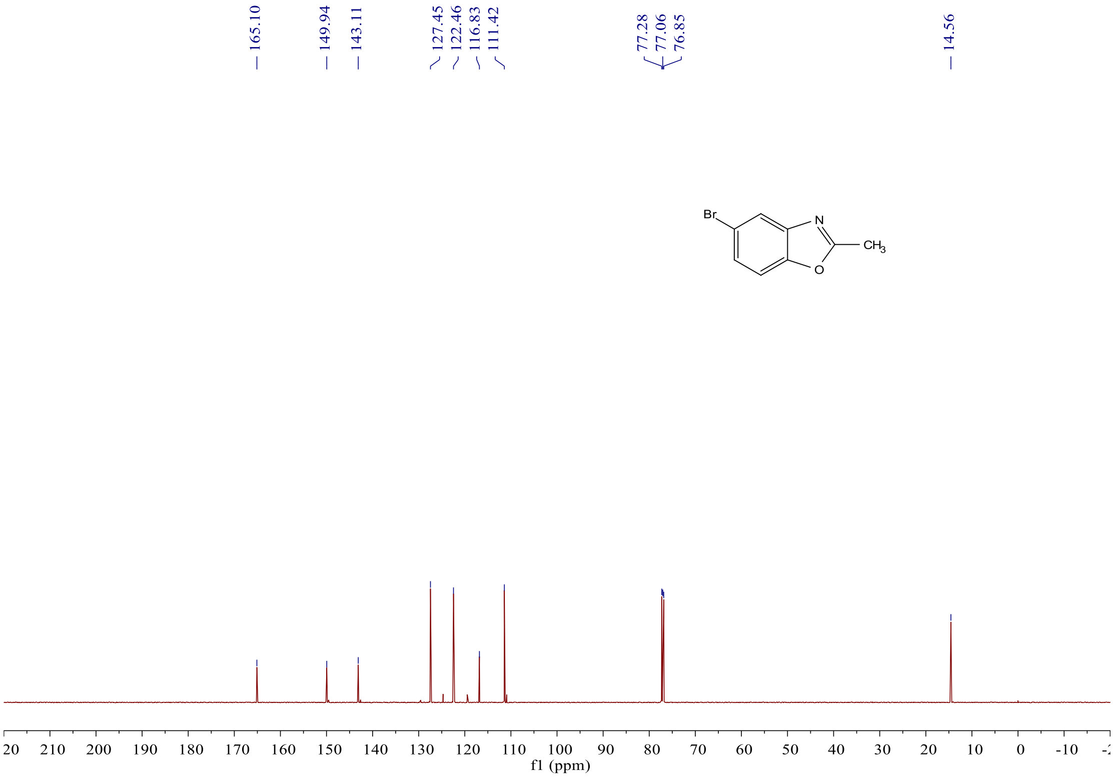

Figure16 ^{13}C NMR spectra of 5-bromo-2-methylbenzo[d]oxazole(**2h**) (solvent $CDCl_3$)

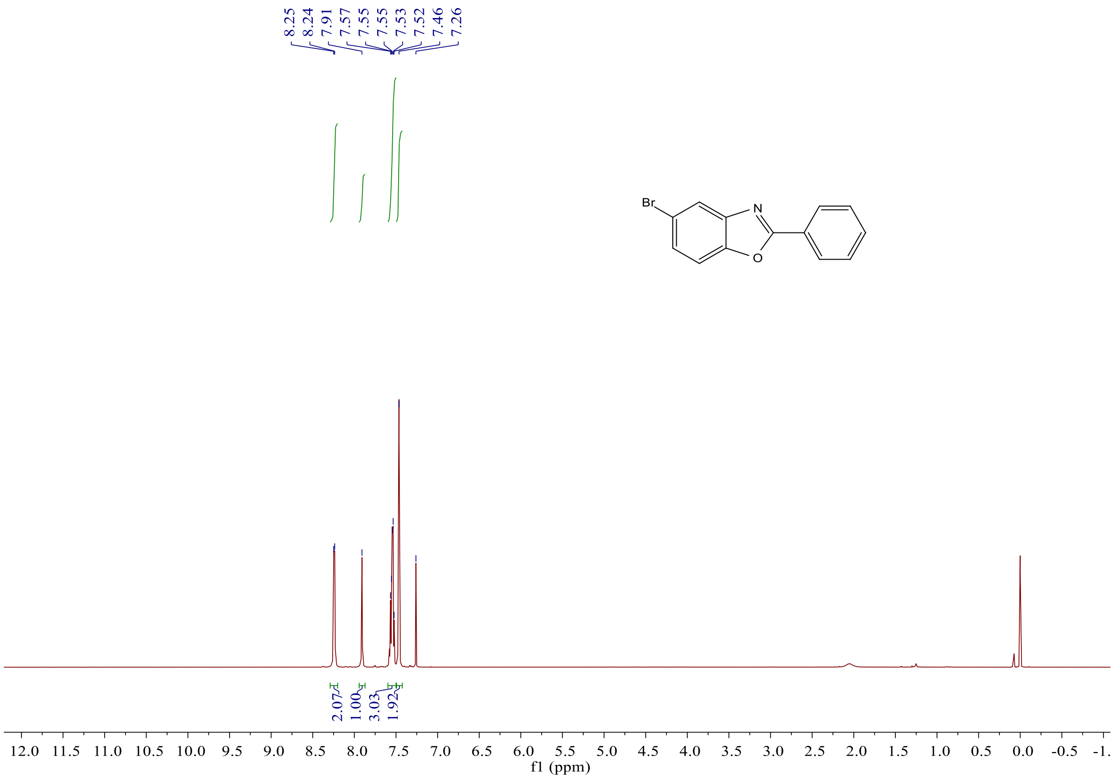

Figure17 ^{1}H NMR spectra of 5-bromo-2-phenylbenzo[d]oxazole(**2i**) (solvent $CDCl_3$)

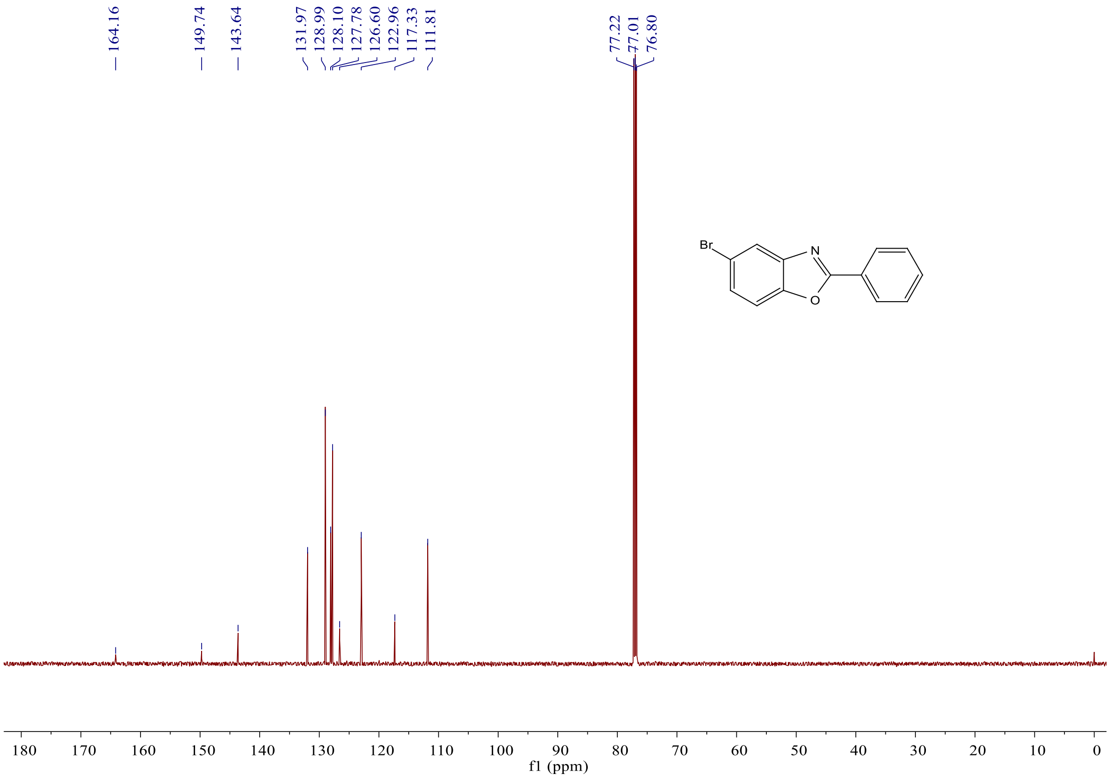

Figure18 ^{13}C NMR spectra of 5-bromo-2-phenylbenzo[d]oxazole(**2i**) (solvent $CDCl_3$)

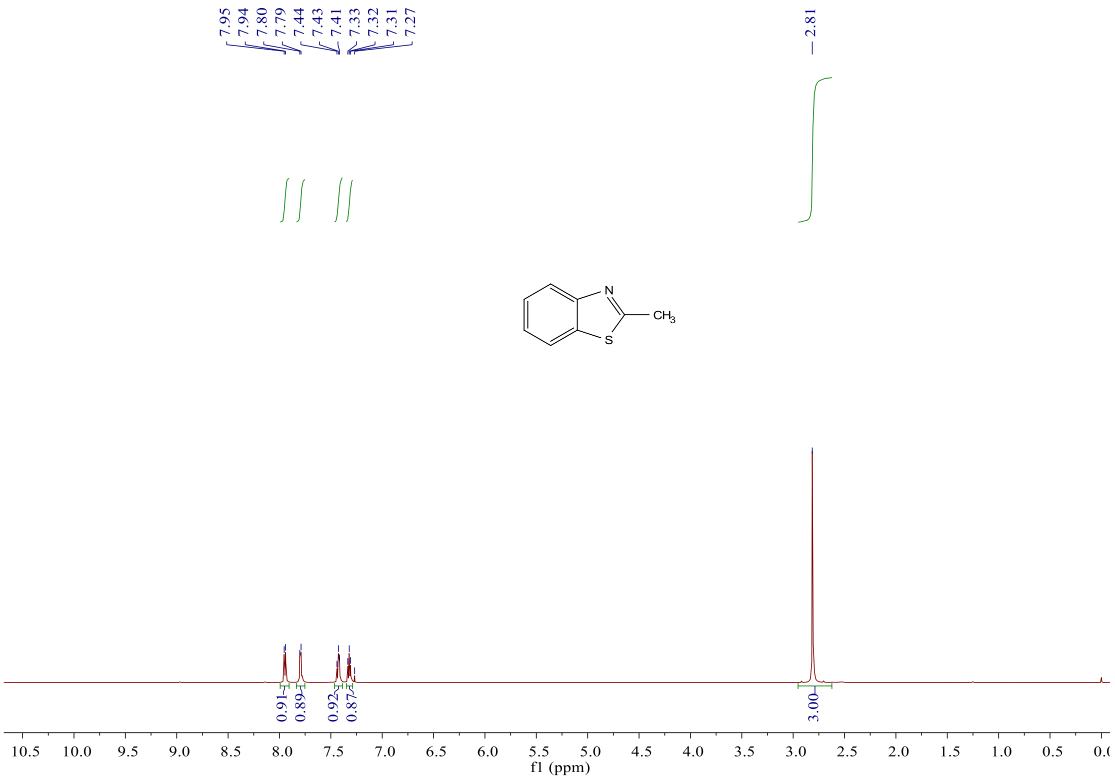

Figure19 ^{1}H NMR spectra of 2-methylbenzo[d]thiazole(**4a**) (solvent $CDCl_3$)

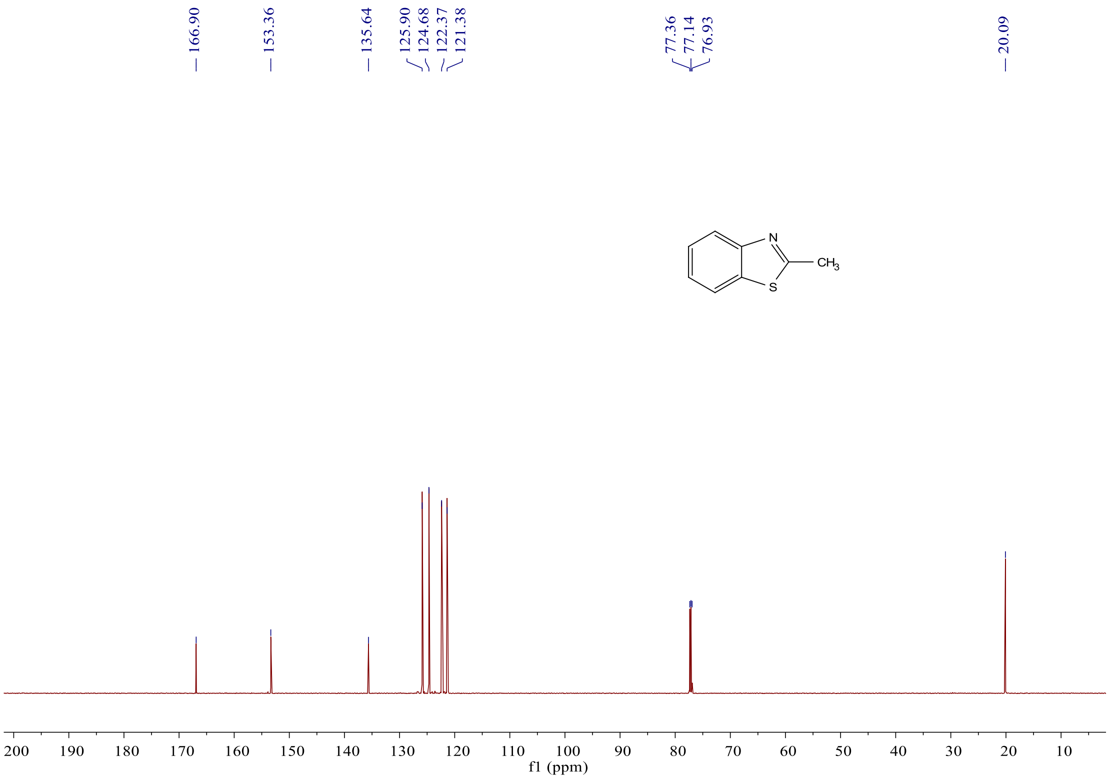

Figure20 ^{13}C NMR spectra of 2-methylbenzo[d]thiazole(**4a**) (solvent $CDCl_3$)

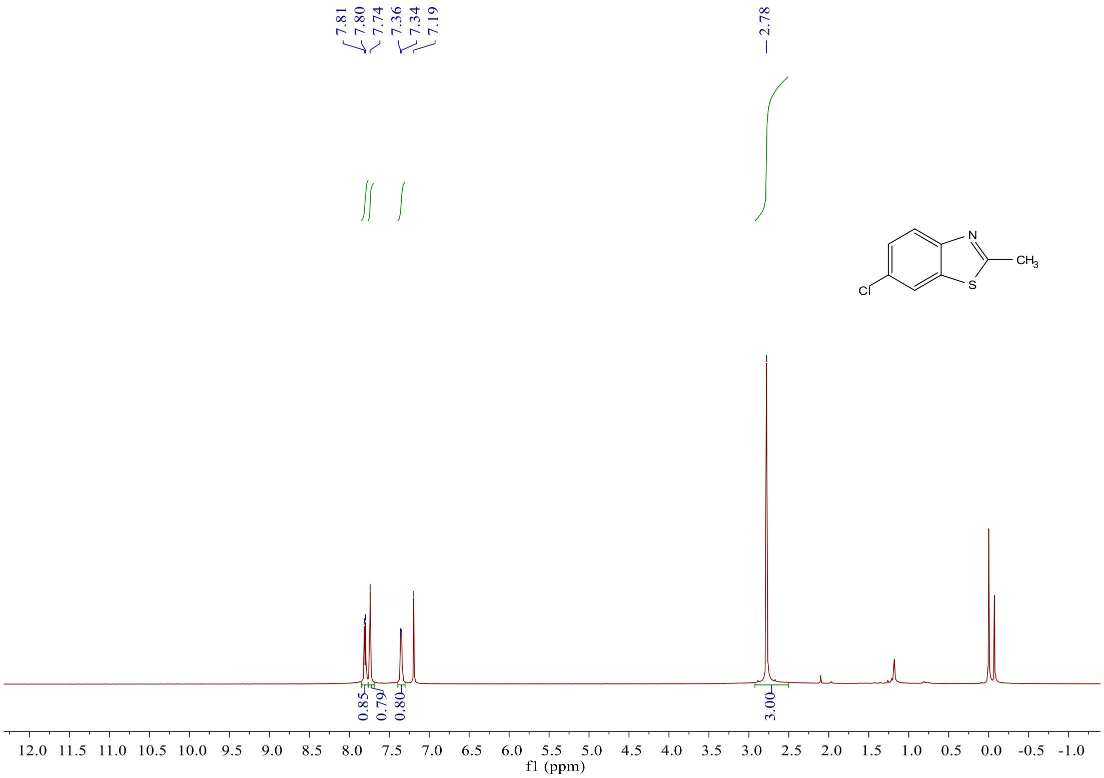

Figure21 ^{1}H NMR spectra of 6-chloro-2-methylbenzo[d]thiazole(**4b**) (solvent $CDCl_3$)

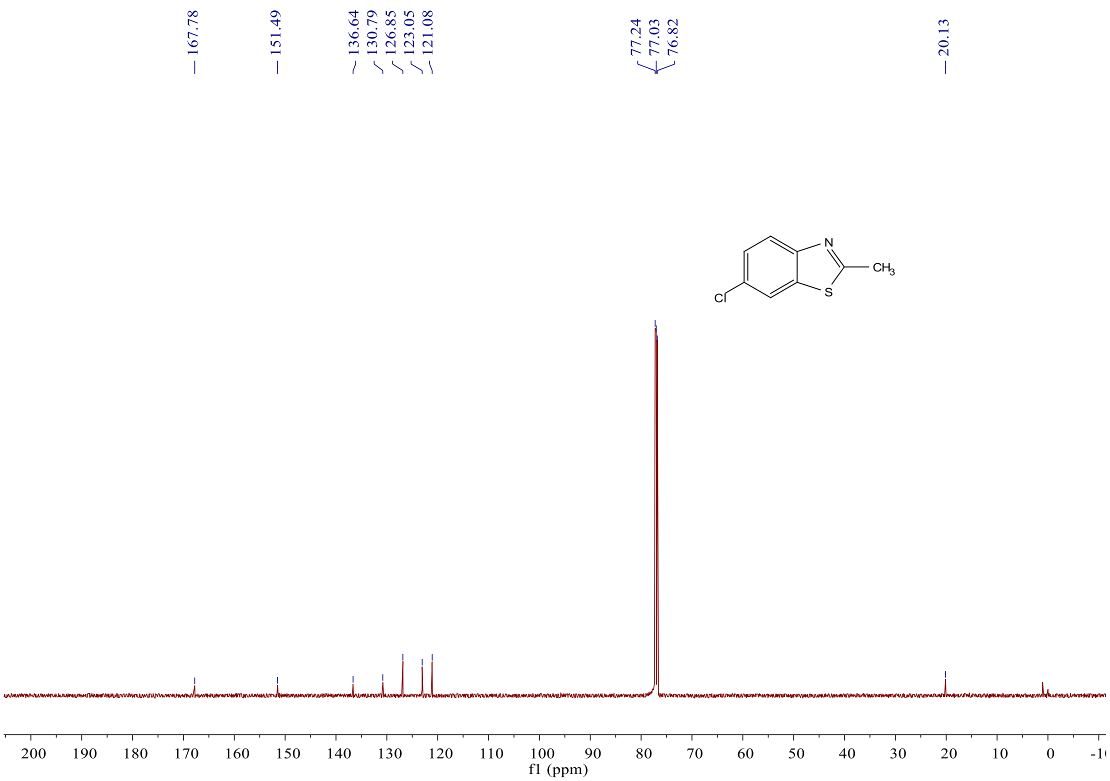

Figure22 ^{13}C NMR spectra of 6-chloro-2-methylbenzo[d]thiazole(**4b**) (solvent $CDCl_3$)

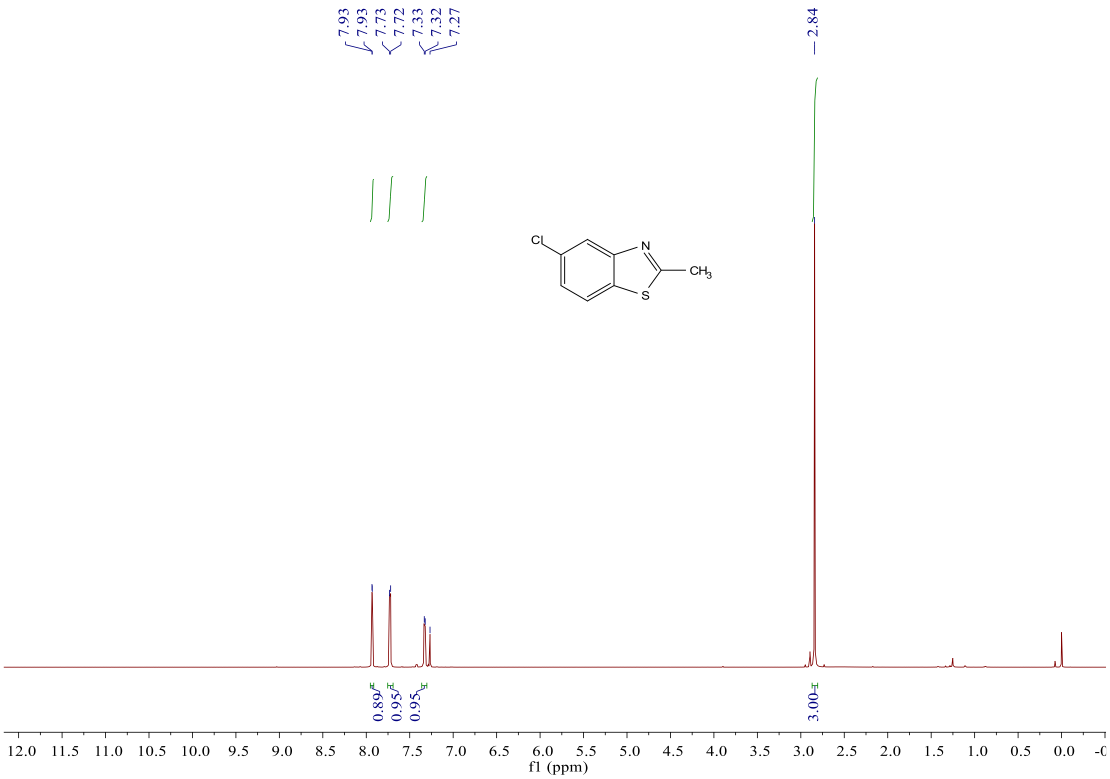

Figure23 ^{1}H NMR spectra of 5-chloro-2-methylbenzo[d]thiazole(**4c**) (solvent $CDCl_3$)

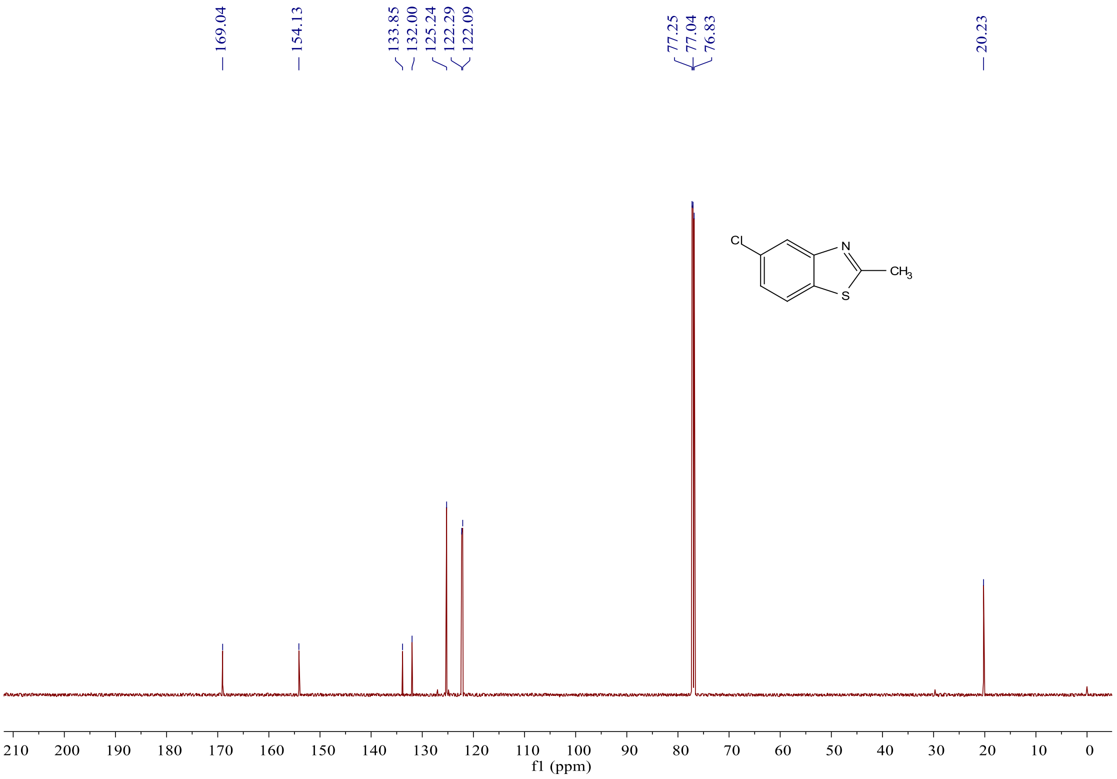

Figure24 ^{13}C NMR spectra of 5-chloro-2-methylbenzo[d]thiazole(**4c**) (solvent $CDCl_3$)

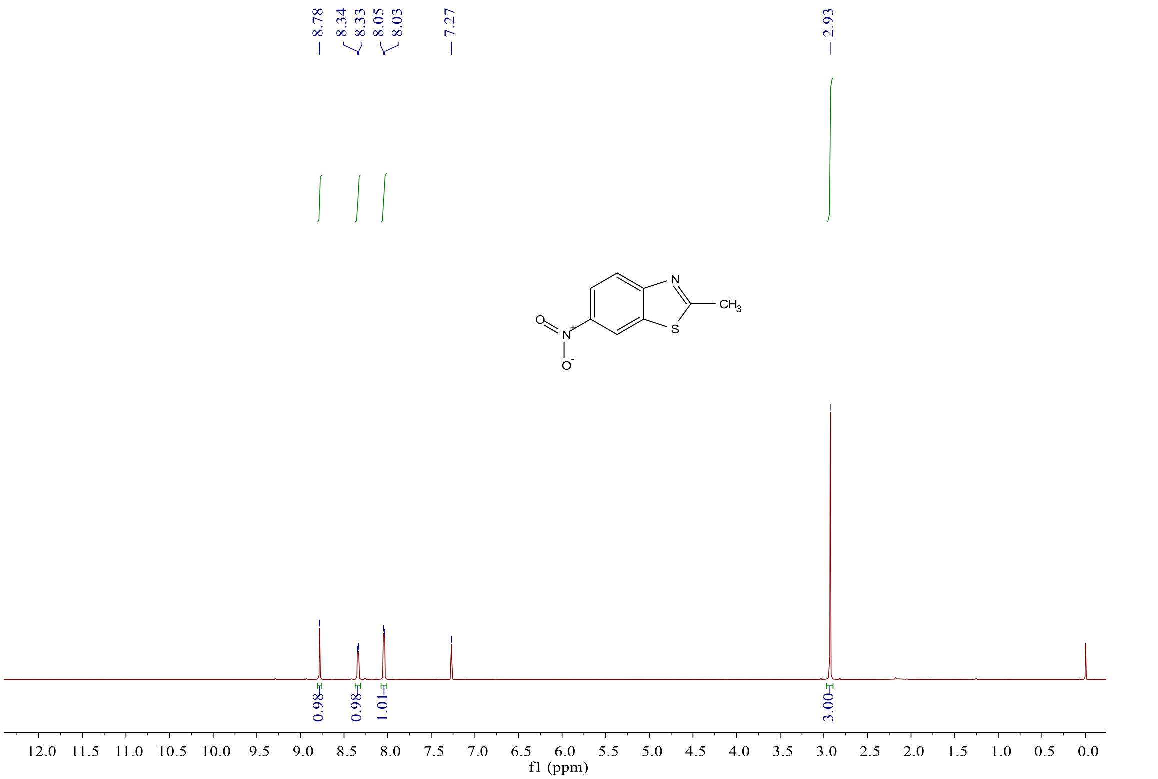

Figure25 ^{1}H NMR spectra of 2-methyl-6-nitrobenzo[d]thiazole(**4d**) (solvent $CDCl_3$)

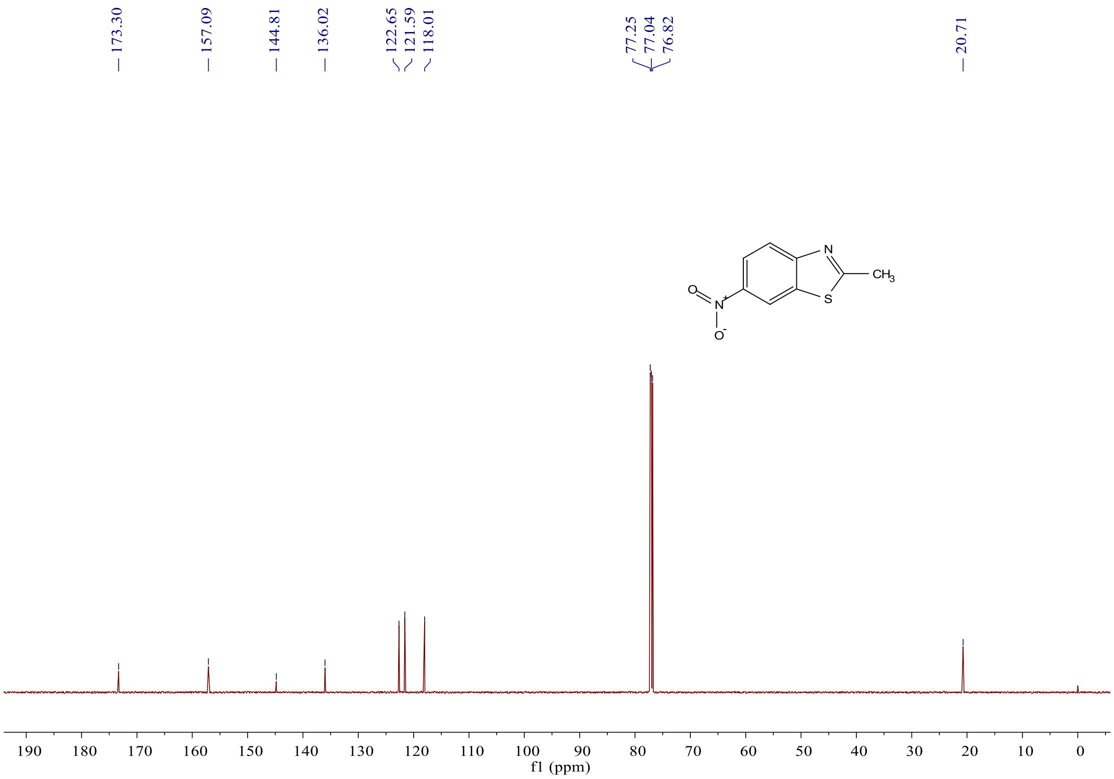

Figure26 ^{13}C NMR spectra of 2-methyl-6-nitrobenzo[d]thiazole(**4d**) (solvent $CDCl_3$)

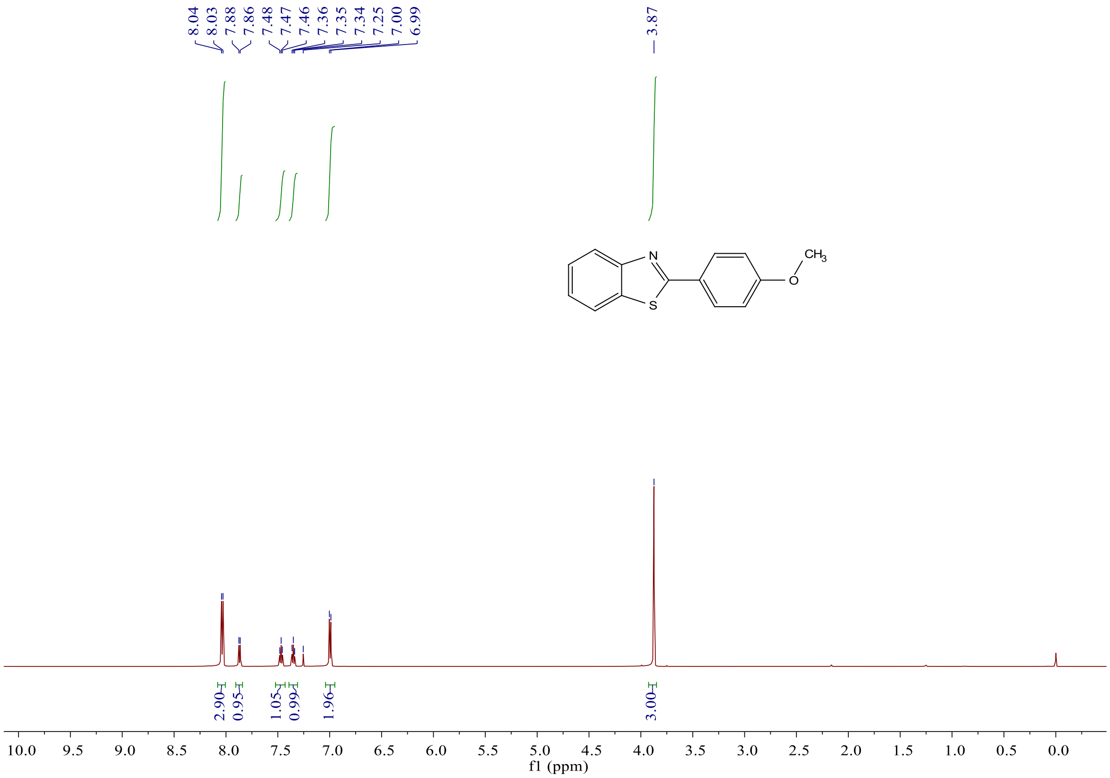

Figure27 ^{1}H NMR spectra of 2-(4-methoxyphenyl)benzo[d]thiazole(**4e**) (solvent $CDCl_3$)

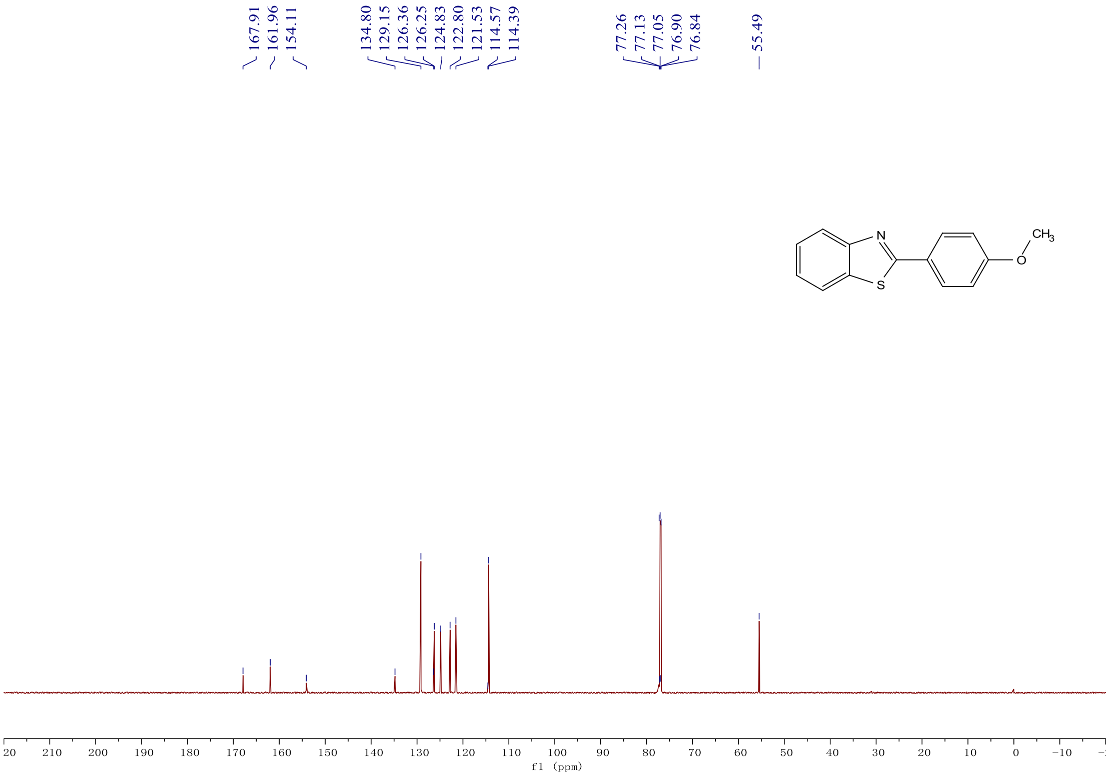

Figure28 ^{13}C NMR spectra of 2-(4-methoxyphenyl)benzo[d]thiazole(**4e**) (solvent $CDCl_3$)

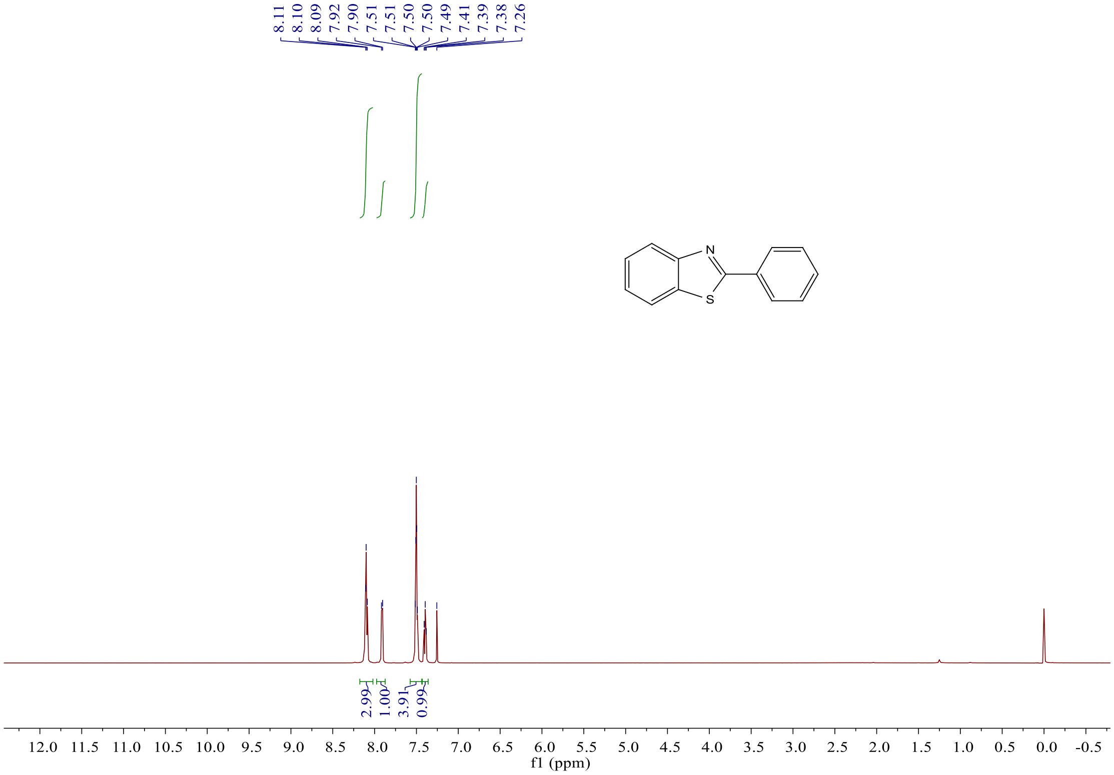

Figure29 ^{1}H NMR spectra of 2-phenylbenzo[d]thiazole (**4f**) (solvent $CDCl_3$)

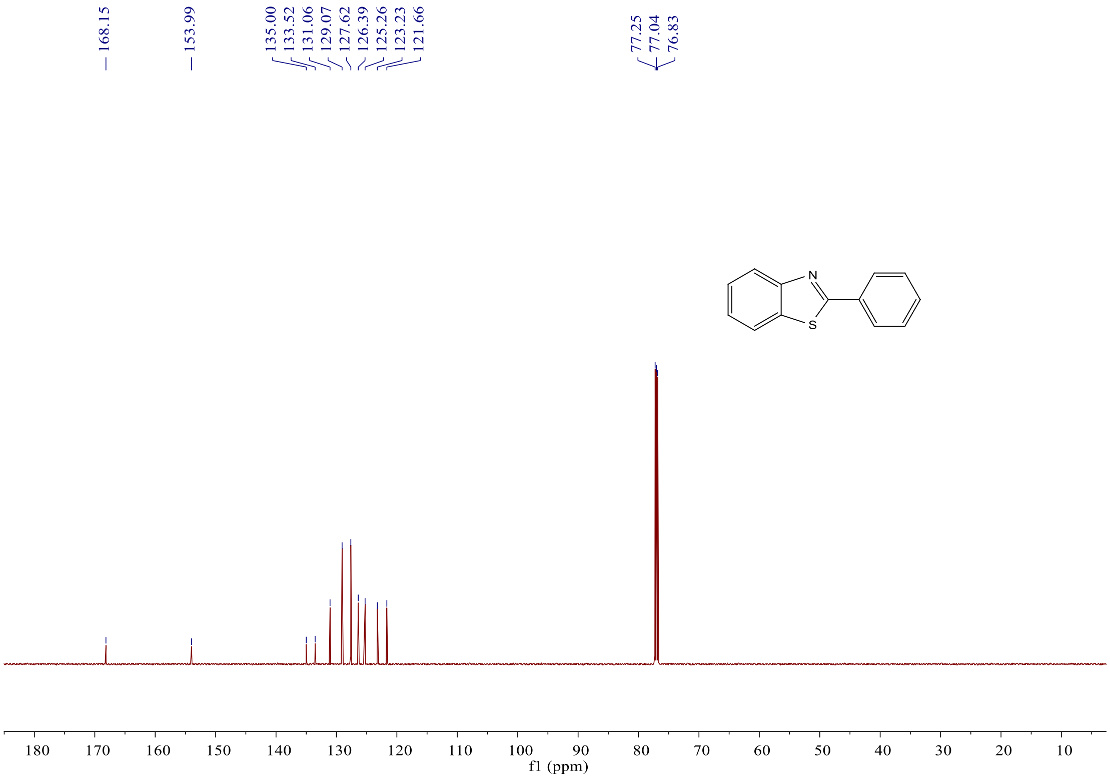

Figure30 ^{13}C NMR spectra of 2-phenylbenzo[d]thiazole (**4f**) (solvent $CDCl_3$)

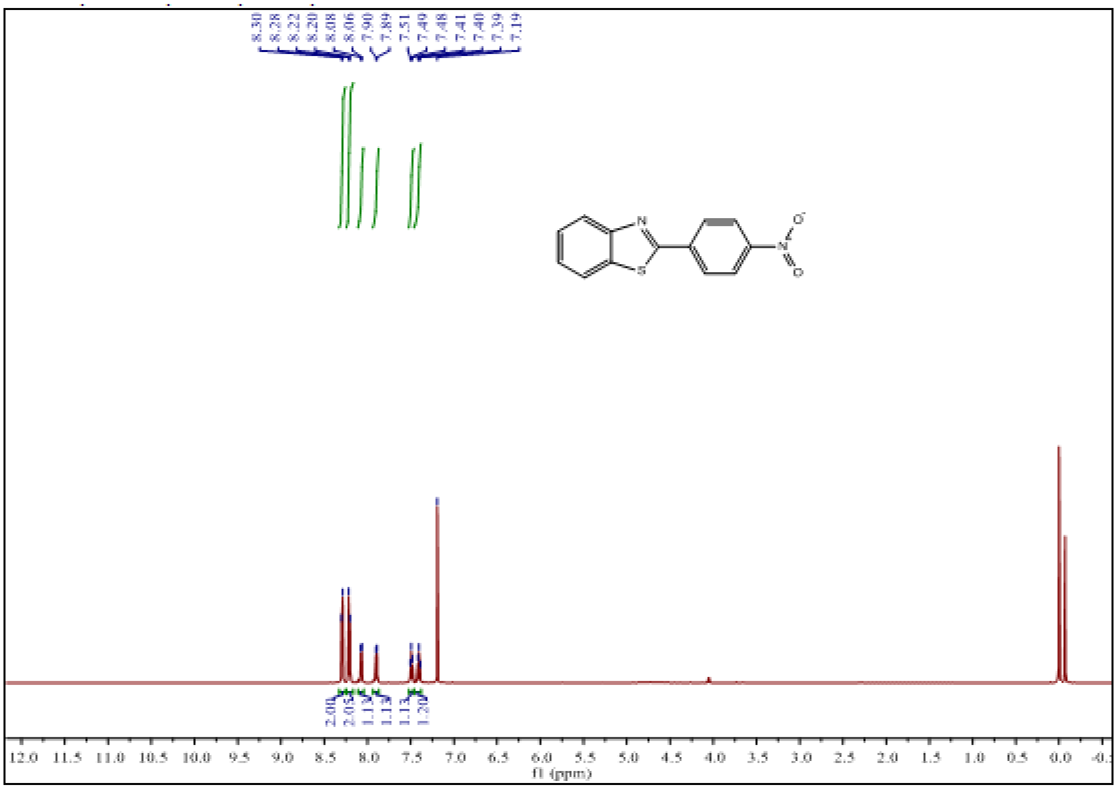

Figure31 ^{1}H NMR spectra of 2-(4-nitrophenyl)benzo[d]thiazole(**4g**) (solvent $CDCl_3$)

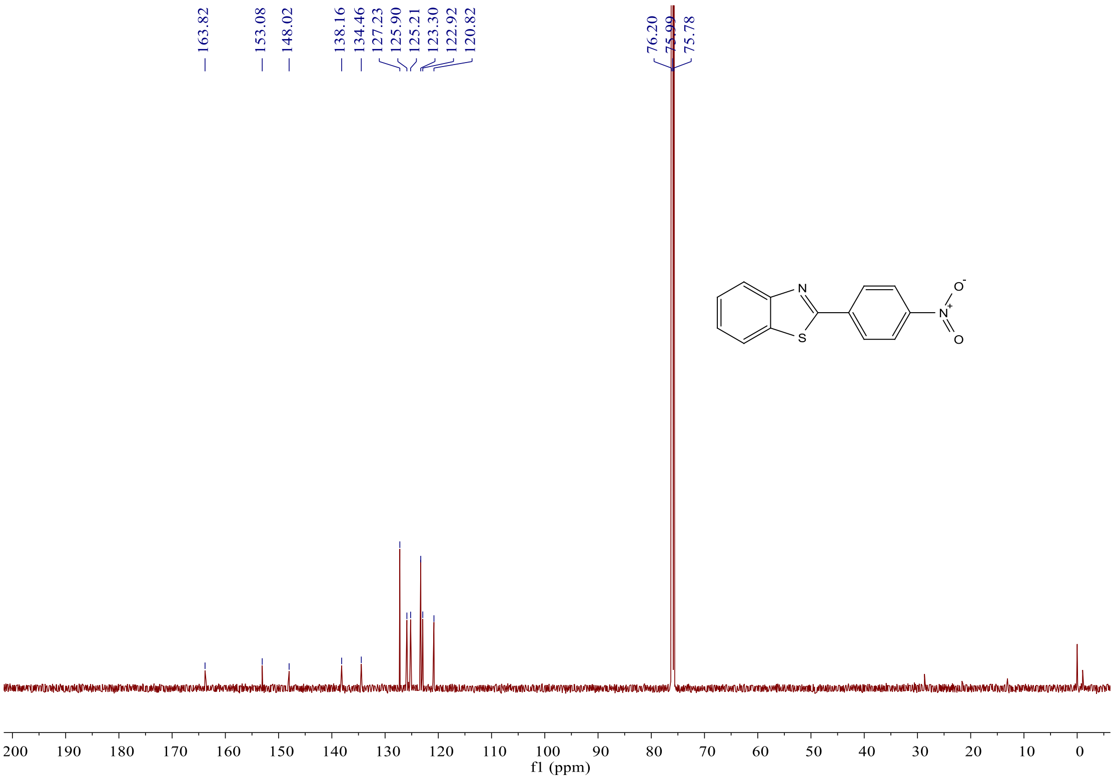

Figure32 ^{13}C NMR spectra of 2-(4-nitrophenyl)benzo[d]thiazole(**4g**) (solvent $CDCl_3$)

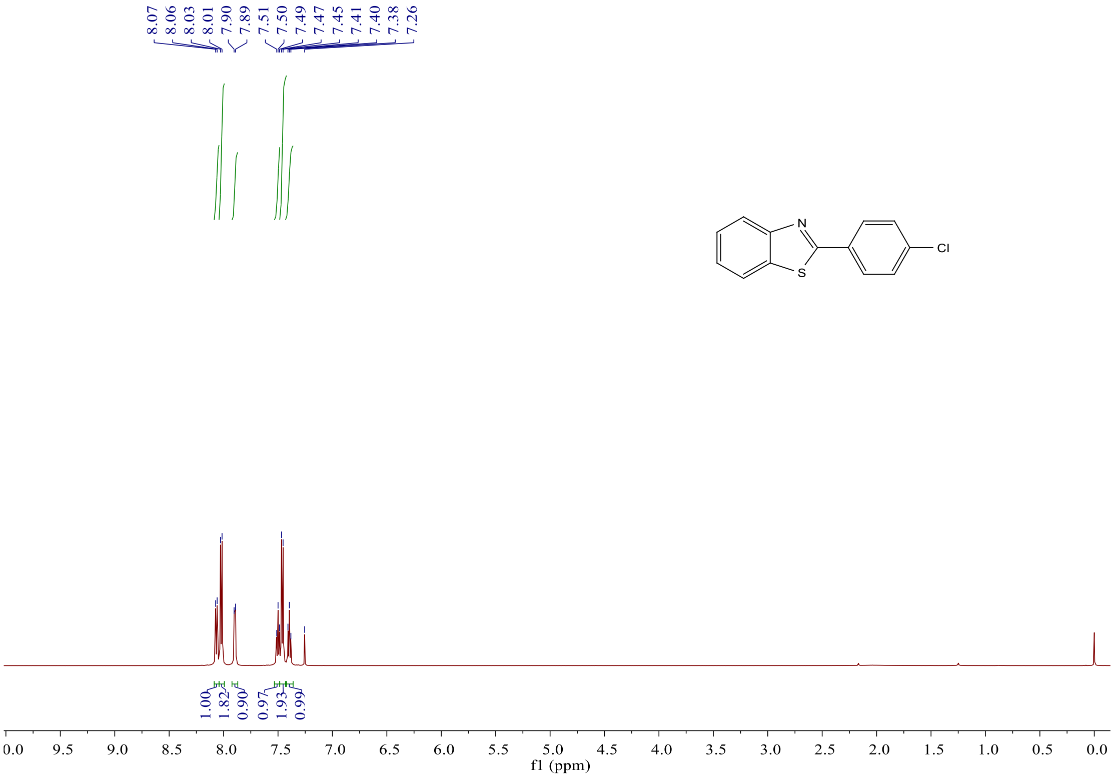

Figure33 ^{1}H NMR spectra of 2-(4-chlorophenyl)benzo[d]thiazole (**4h**) (solvent $CDCl_3$)

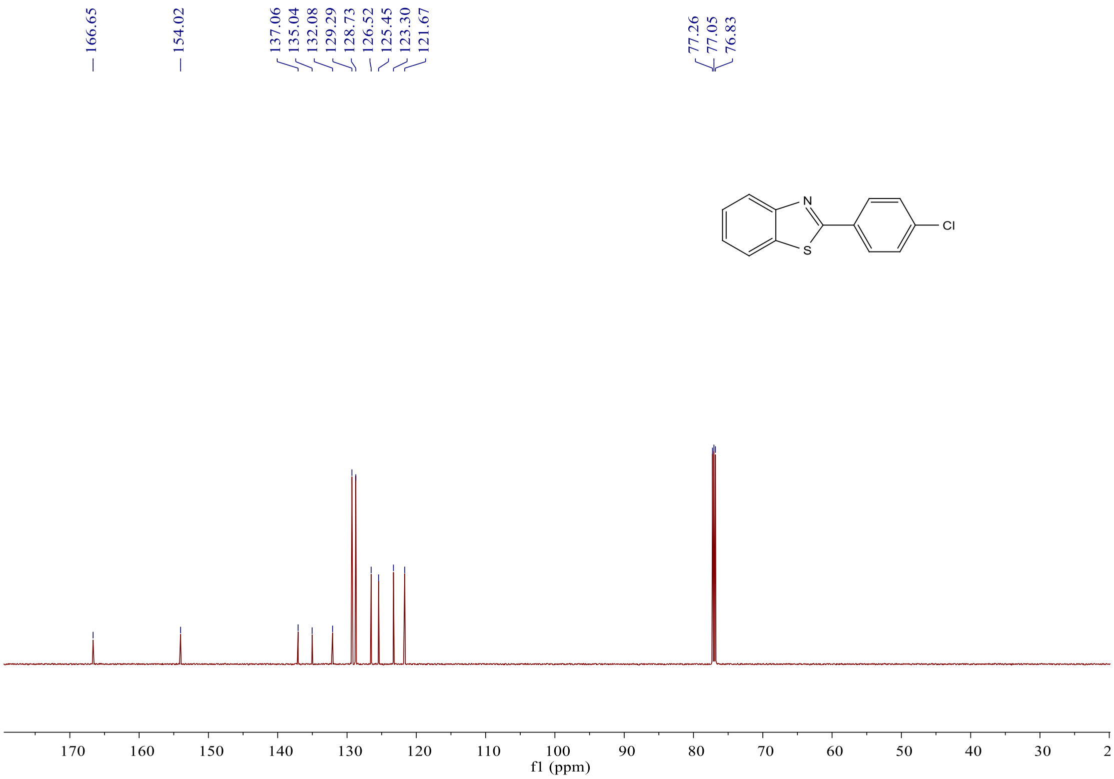

Figure34 ^{13}C NMR spectra of 2-(4-chlorophenyl)benzo[d]thiazole (**4h**) (solvent $CDCl_3$)

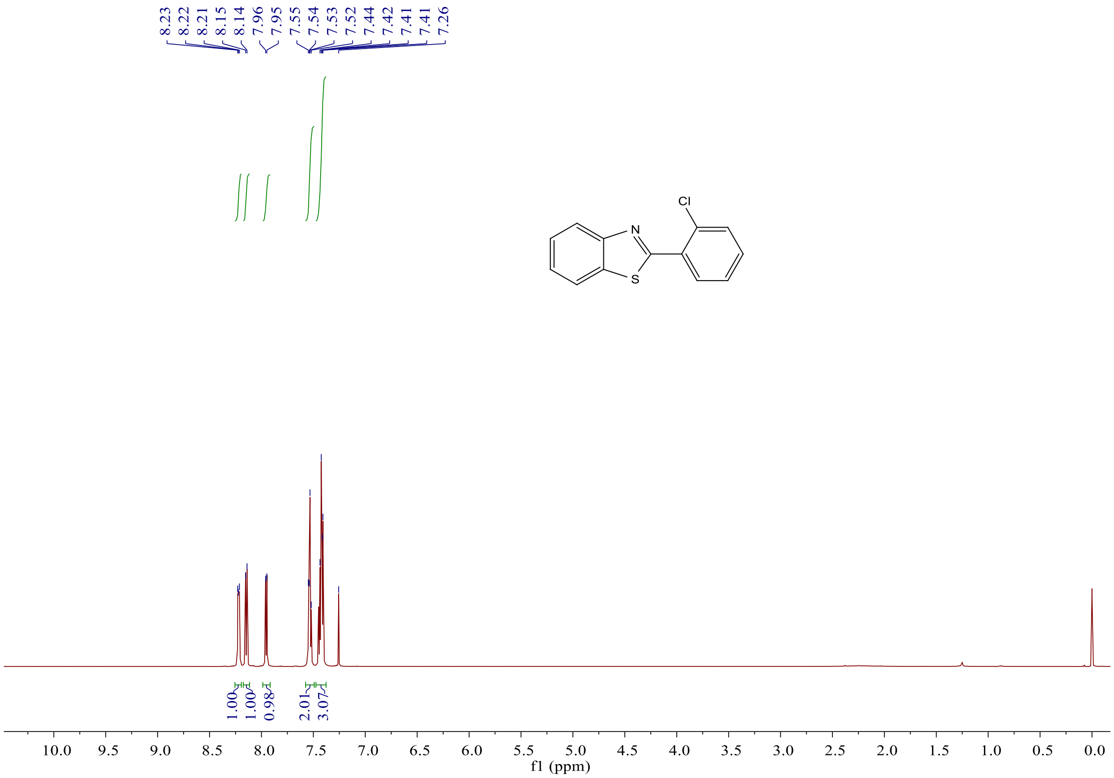

Figure35 ^{1}H NMR spectra of 2-(2-chlorophenyl)benzo[d]thiazole(**4i**) (solvent $CDCl_3$)

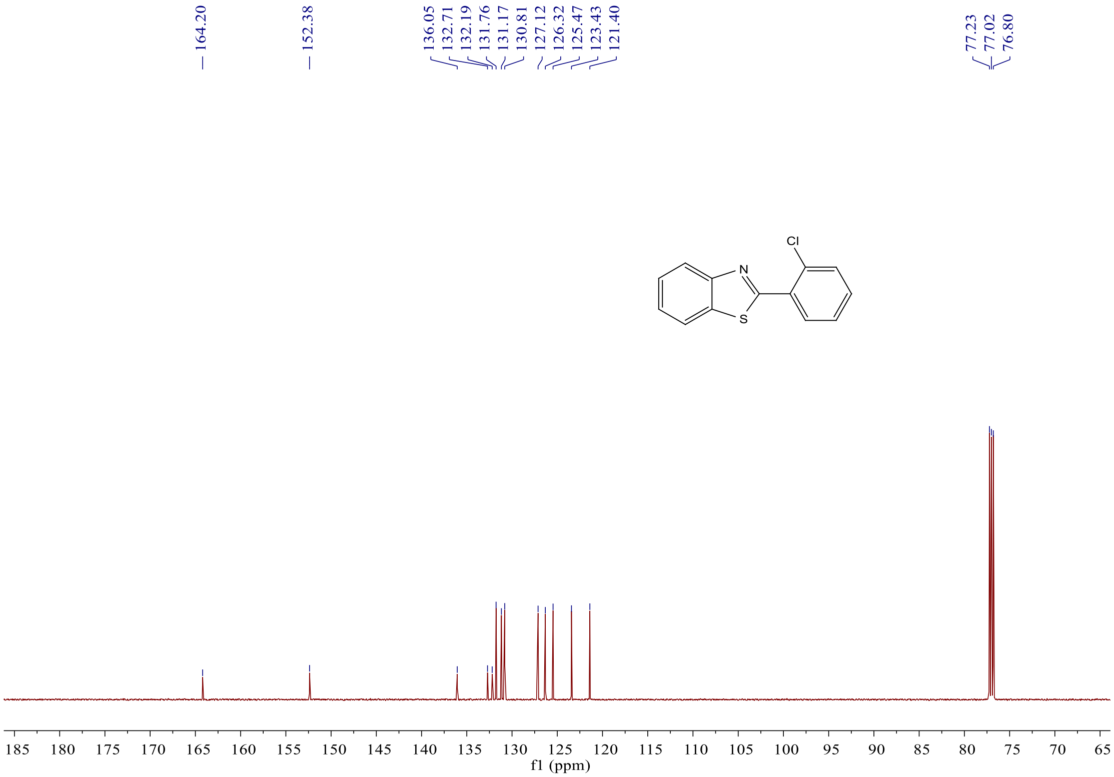

Figure36 ^{13}C NMR spectra of 2-(2-chlorophenyl)benzo[d]thiazole(**4i**) (solvent $CDCl_3$)

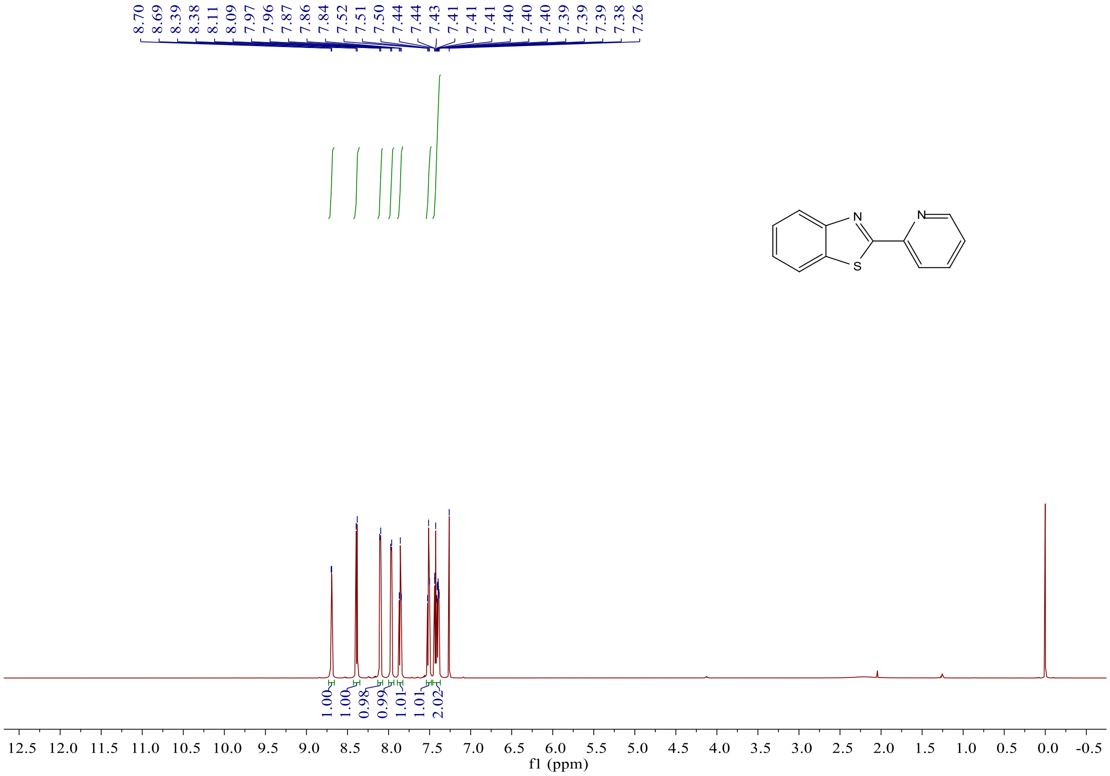

Figure37 ^{1}H NMR spectra of 2-(pyridin-2-yl)benzo[d]thiazole(**4j**) (solvent $CDCl_3$)

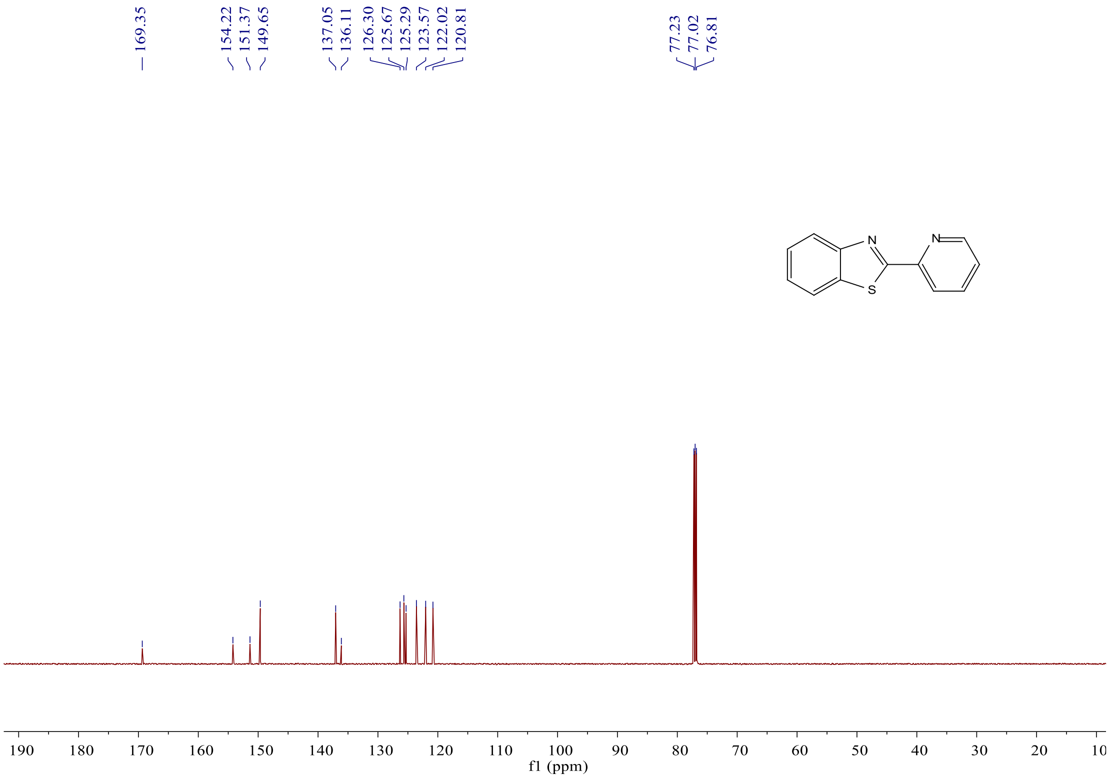

Figure38 ^{13}C NMR spectra of 2-(pyridin-2-yl)benzo[d]thiazole(**4j**) (solvent $CDCl_3$)

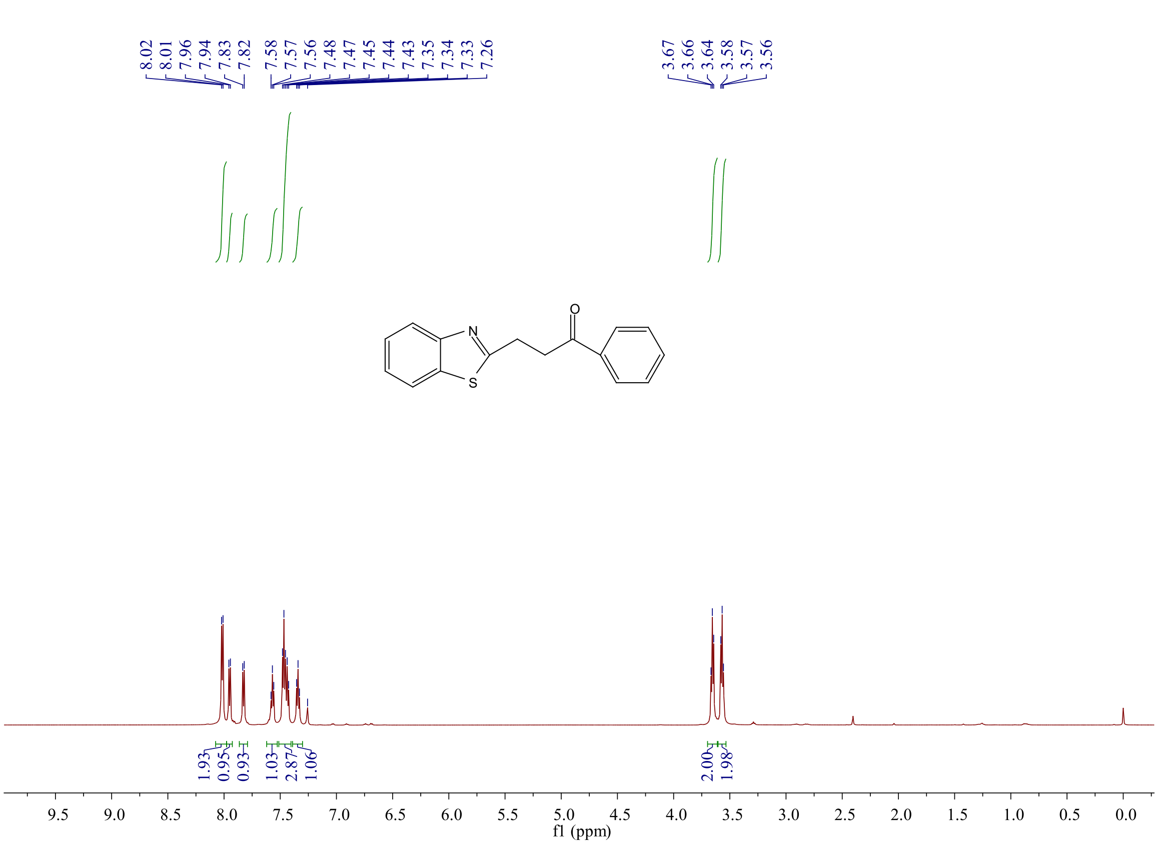

Figure39 ¹H NMR spectra of 3-Benzothiazol-2-yl-1-phenyl-propan-1-one (**4k**) (solvent $CDCl_3$)

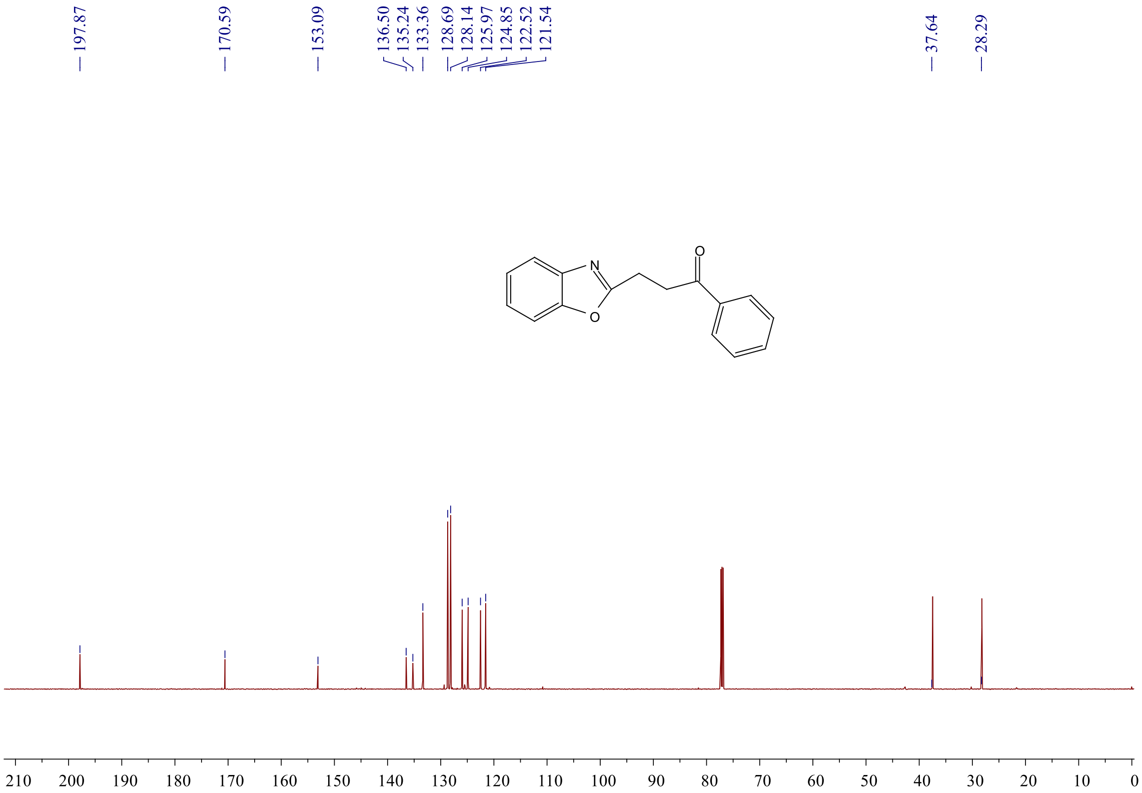

Figure40 ^{13}C NMR spectra of 3-Benzothiazol-2-yl-1-phenyl-propan-1-one (**4k**) (solvent $CDCl_3$)
